# Using singular perturbation theory to determine kinetic parameters in a non-standard coupled enzyme assay

**DOI:** 10.1007/s00285-020-01524-8

**Published:** 2020-08-06

**Authors:** Mohit P. Dalwadi, Diego Orol, Frederik Walter, Nigel P. Minton, John R. King, Katalin Kovács

**Affiliations:** 1 Mathematical Institute, University of Oxford, Radcliffe Observatory Quarter, Oxford, OX2 6GG UK; 2 Synthetic Biology Research Centre, University of Nottingham, University Park, Nottingham, NG7 2RD UK; 3 School of Mathematical Sciences, University of Nottingham, University Park, Nottingham, NG7 2RD UK

**Keywords:** Asymptotic analysis, Reaction kinetics, Synthetic biology, Enzyme characterization, Aminotransaminase, 92C45, 92C42, 34E10, 34E15, 37N25

## Abstract

We investigate how to characterize the kinetic parameters of an aminotransaminase using a non-standard coupled (or auxiliary) enzyme assay, where the peculiarity arises for two reasons. First, one of the products of the auxiliary enzyme is a substrate for the primary enzyme and, second, we explicitly account for the reversibility of the auxiliary enzyme reaction. Using singular perturbation theory, we characterize the two distinguished asymptotic limits in terms of the strength of the reverse reaction, which allows us to determine how to deduce the kinetic parameters of the primary enzyme for a characterized auxiliary enzyme. This establishes a parameter-estimation algorithm that is applicable more generally to similar reaction networks. We demonstrate the applicability of our theory by performing enzyme assays to characterize a novel putative aminotransaminase enzyme, CnAptA (UniProtKB Q0KEZ8) from Cupriavidus necator H16, for two different omega-amino acid substrates.

## Introduction

Enzyme assays are an important tool for characterizing enzymes. In the classic assay, a single reaction converts a substrate into a product, using an enzyme as a catalyst, and the product is measured over time to estimate the initial reaction velocity (Bisswanger 2014). A mathematical analysis allows one to use this information to deduce the kinetic properties of the enzyme in question (Murray 2002). When characterizing an enzyme for which the reaction product is difficult to observe, an auxiliary enzyme can be introduced to convert the product of the primary enzyme reaction into a chemical that can more easily be measured (*e.g.* NADH) (Storer and Cornish-Bowden 1974; Rudolph et al. 1979). This is called a coupled enzyme assay. However, the problem is more complicated if the reaction catalysed by the auxiliary enzyme has a product which is also a substrate for the primary enzyme.

**Fig. 1:**
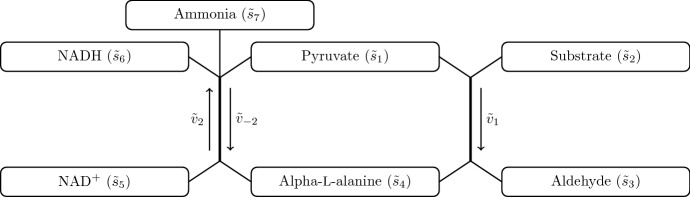
A diagram of the reaction network we investigate in this paper. Each box denotes a chemical compound involved in the network, and each arrow denotes a reaction to and from the specified group of products and substrates, respectively. Each reaction is catalysed by an enzyme; the primary enzyme catalyses the reaction on the right, marked with velocity ${\tilde{v}}_{1}$, and the auxiliary (secondary) enzyme catalyses both directions of the reversible reaction on the left, marked with forward velocity ${\tilde{v}}_{2}$ and backwards velocity ${\tilde{v}}_{-2}$. Note that our model does not rely on pyruvate being converted into alpha-L-alanine, it being valid for the primary enzyme being a general aminotransaminase. Hence, in this diagram ‘pyruvate’ can be generalized to ‘keto acid’, and ‘alpha-L-alanine’ can be generalized to ‘amino acid’

In this paper, we are interested in understanding how to characterize a primary enzyme in a non-standard coupled enzyme assay, where one of the products of the auxiliary enzyme is a substrate for the primary enzyme and where we explicitly account for the reversibility of the auxiliary enzyme reaction. This is motivated by the characterization of the class of enzymes known as *aminotransaminases*, which catalyse the transfer of keto and amine groups between organic compounds (Slabu et al. 2017), and are an important class of biocatalysts in synthetic biology for reasons we discuss below. In Fig. 1 we show a schematic of the reaction network we consider in this paper. While we refer to the specific organic compounds pyruvate and alpha-L-alanine in Fig. 1 and through the paper in order to be consistent with the experiments we perform, we emphasize that our analysis is more general. In particular, the general organic-group-transfer property of aminotransaminases means that ‘pyruvate’ can be generalized to ‘keto acid’, and ‘alpha-L-alanine’ can be generalized to ‘amino acid’ for an aminotransaminase primary enzyme. Since ammonia is produced when an amino acid is dehydrogenated, the presence of ammonia is a consequence of using an aminotransaminase as the primary enzyme. This means that the reaction network we show in Fig. 1 is particularly applicable to coupled enzyme assays with the primary enzyme being an aminotransaminase.

The technique to deal with a standard coupled enzyme assay with one primary enzyme and one auxiliary enzyme was first outlined within a mathematical framework in McClure (1969). We direct the interested reader to Rudolph et al. (1979) for a history of the theoretical work on standard coupled enzyme assays, as well as a practical guide to performing these types of assays. In this paper, we investigate when the standard approach identified in McClure (1969) is appropriate for the non-standard assay we consider, and how to modify the approach when it is not. We then implement our theory by performing assays on a previously-uncharacterized enzyme for two different substrates.

Calculating enzyme properties through enzyme assays is an inherently dynamic process; the steady state is reached only when one of the substrates vanishes. In this dynamic process, it is helpful to measure the indicator chemical during a regime where the reaction velocity is approximately constant, corresponding to a linear increase of the indicator chemical in time (we henceforth refer to this as a ‘linear growth’ regime). Often this means estimating the reaction velocity through initial rate experiments, with the goal of avoiding complications due to reversible reactions. Linear growth is helpful as a sanity check that things are proceeding as they should (typically using the ‘eyeball norm’). Moreover, experimental noise can increase the error when attempting to implement an accurate fit in nonlinear systems - restricting oneself to a linear growth regime significantly reduces this issue.

For these reasons, we use singular perturbation theory (Bender and Orszag 2013; Kevorkian and Cole 2013) to analyse the mathematical systems we derive, and to understand how to determine the kinetic parameter values of the primary enzyme in such a system. This provides a significant reduction of the complexity of the system, and thus allows us to minimize issues associated with experimental noise, as we aim to determine functional forms for the measurable reaction velocities. Moreover, as exhibited in recent examples (Dalwadi et al. 2018a, b; Kumar and Josić 2011; Eilertsen et al. 2018; Eilertsen and Schnell 2018), singular perturbation theory is particularly well suited to understanding systems of chemical reactions exhibiting a significant separation of timescales, so we will use a similar approach here.

In our analysis, we assume that the timescales of enzyme complex formation are much shorter than the other reaction timescales in our problem, and that the effective reaction velocities are governed by Michaelis–Menten-type laws. The suitability of the Michaelis–Menten equations in many scenarios, including but not limited to standard coupled enzyme assays, has been investigated in recent work by Schnell and colleagues, for example Stroberg and Schnell (2016); Eilertsen et al. (2018); Eilertsen and Schnell (2018), using singular perturbation theory.

To demonstrate how our theoretical results can be used to characterize enzymes, we perform several coupled enzyme assays to characterize a putative omega-aminotransferase (also known as an aminotransaminase or an omega-amino-acid amino transferase) enzyme, CnAptA, for two omega-amino acid substrates. Omega-aminotransferases belong to class III aminotransferases; these catalyse the transamination of omega-amino acids such as beta-alanine or gamma-aminobutyric acid by transferring the amino group to a keto acid, using pyridoxal 5$'$-phosphate (PLP) as a cofactor (Mehta et al. 1993). Aminotransferases are increasingly important biocatalysts for the synthesis of industrially relevant chiral compounds and in the pharmaceutical industry for the production of optically pure amines and amino alcohols necessary for the synthesis of important drugs (Sayer et al. 2013). Moreover, aminotransferases are particularly important in synthetic biology because they are the key to creating biosustainable production routes to many important platform chemicals. That is, carboxylic acids make up eight of the top twelve platform chemicals selected by the US Department of Energy that can be derived from biomass (Werpy and Petersen 2004). As current industrial methods to produce these acids often involve fossil fuels, biological production routes provide environmentally sustainable alternatives.

The outline of this paper is as follows. In Sect. 2, we briefly recap the mathematical analysis of the standard enzyme assay, which allows us to put the main results of this paper into an appropriate context. In Sect. 3, we derive a mathematical model for the non-standard coupled enzyme assay of interest in this paper, which has the reaction network shown in Fig. 1. We then explore how the non-standard assay differs from the standard one and determine how the measured reaction velocity relates to the kinetic parameters of the primary enzyme in the non-standard case. We do this using an asymptotic analysis to systematically reduce the complexity of the system, and we present the main distinguished limit (*i.e.* the dominant balance of most practical relevance) of the system in Sect. 3.1. We present the remaining distinguished limit in Sect. 3.2, where we find that the distinguished nature of this limit is apparent only in a non-measurable chemical during the linear regime or during the depletion regime, and that the measurable system behaviour in the linear regime is captured by a sub-limit of the strong reverse reaction regime treated in Sect. 3.1. We summarise our theoretical results in Sect. 4, outlining the procedure that should be implemented to use experimental data to infer the kinetic parameters of the primary enzyme. In the same section, we compare our proposed method with a naive nonlinear fit, verifying that our method is preferable. In Sect. 5, we demonstrate that our results can be used effectively in characterizing enzymes by performing the coupled enzyme assay modelled in this paper and applying our theoretical results. Finally, in Sect. 6, we discuss our results in the context of their significance for experimental approaches involving non-standard coupled enzyme assays.

## Standard coupled enzyme assay

**Fig. 2:**
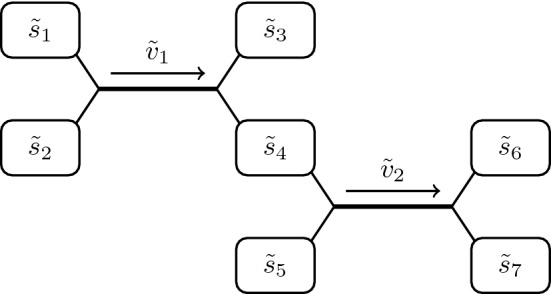
Abstract network diagram for a standard enzyme assay. The primary enzyme has reaction velocity ${\tilde{v}}_{1}$ and the auxiliary enzyme has reaction velocity ${\tilde{v}}_{2}$

We start by briefly recapping the mathematical model for the standard coupled enzyme assay, presented in broad dimensional terms in McClure (1969). In this section, we present the relevant chemicals involved in abstract terms before relating them to the chemicals labelled in Fig. 1 for the non-standard coupled enzyme assay we consider in the next section. Our goal in this section is to recap how to determine the kinetic parameters of the primary enzyme in the system shown in Fig. 2, where we assume knowledge of the kinetic parameters of the auxiliary enzyme. Experimentally, we are able to measure the concentration of ${\tilde{s}}_6$, and so our overarching goal is to understand how to use this measurement to infer the kinetic parameters that characterize the primary enzyme.

In general, the kinetic parameters used to characterize an enzyme are intrinsic to the ordinary differential equations (ODEs) assumed to govern this system, using Michaelis–Menten-type laws to quantify each reaction velocity (Menten and Michaelis 1913). The network in Fig. 2 implies the following seven dimensional ODEs

1 $$\begin{aligned}&\dfrac{\mathrm {d} {\tilde{s}}_1}{\mathrm {d} {\tilde{t}}} = - {\tilde{v}}_{1}, \quad \dfrac{\mathrm {d} {\tilde{s}}_2}{\mathrm {d} {\tilde{t}}} = - {\tilde{v}}_{1}, \quad \dfrac{\mathrm {d} {\tilde{s}}_3}{\mathrm {d} {\tilde{t}}} = {\tilde{v}}_{1}, \quad \dfrac{\mathrm {d} {\tilde{s}}_4}{\mathrm {d} {\tilde{t}}} = {\tilde{v}}_{1}- {\tilde{v}}_{2}, \quad \nonumber \\&\qquad \quad \qquad \dfrac{\mathrm {d} {\tilde{s}}_5}{\mathrm {d} {\tilde{t}}} {=} - {\tilde{v}}_{2}, \dfrac{\mathrm {d} {\tilde{s}}_6}{\mathrm {d} {\tilde{t}}} {=} {\tilde{v}}_{2}, \dfrac{\mathrm {d} {\tilde{s}}_7}{\mathrm {d} {\tilde{t}}} = {\tilde{v}}_{2}. \end{aligned}$$

We use the convention that dimensional quantities are marked with a tilde, whereas dimensionless quantities are not. The initial conditions are

2 $$\begin{aligned}&{\tilde{s}}_1(0) = {\tilde{\alpha }}, \quad {\tilde{s}}_2(0) = {\tilde{\beta }}, \quad {\tilde{s}}_3(0) = 0, \quad {\tilde{s}}_4(0) = 0,\nonumber \\ {}&\qquad {\tilde{s}}_5(0) = {\tilde{\gamma }}, \quad {\tilde{s}}_6(0) = 0, {\tilde{s}}_7(0) = 0, \end{aligned}$$

which corresponds to a system initially containing the minimal number of substrates necessary for both reactions to take place: ${\tilde{s}}_1$, ${\tilde{s}}_2$, and ${\tilde{s}}_5$, then instantaneously adding the primary and auxiliary enzyme such that the entire system is well-mixed. We assume that the affinity a given substrate has for an enzyme is independent of the other substrates. Therefore, the reaction velocities follow Michaelis–Menten kinetics generalized to multiple substrates (Alberty 1953)

3 $$\begin{aligned} {\tilde{v}}_{1}= {\tilde{k}}^{(1)}\left( \dfrac{{\tilde{s}}_1}{{\tilde{K}}_1^{(1)}+ {\tilde{s}}_1}\right) \left( \dfrac{{\tilde{s}}_2}{{\tilde{K}}_2^{(1)}+ {\tilde{s}}_2}\right) , \quad {\tilde{v}}_{2}= {\tilde{k}}^{(2)}\left( \dfrac{{\tilde{s}}_4}{{\tilde{K}}_4^{(2)}+ {\tilde{s}}_4}\right) \left( \dfrac{{\tilde{s}}_5}{{\tilde{K}}_5^{(2)}+ {\tilde{s}}_5}\right) . \end{aligned}$$

This problem was recently investigated through a singular perturbation analysis in Eilertsen and Schnell (2018) to determine when the Michaelis–Menten reaction-type formulation of the problem is valid by considering the intermediate enzyme complexes formed during each reaction. We will assume throughout this paper that we are in this regime, essentially assuming the reactant-stationary assumption holds, *i.e.* that the primary substrates are approximately constant as the primary intermediate complexes form.

In (3), we use the convention that a subscript refers to a relationship with a particular chemical, and a bracketed superscript refers to a particular reaction. For example, the parameter ${\tilde{K}}_4^{(2)}$ refers to the Michaelis constant for ${\tilde{s}}_4$ in the reaction velocity ${\tilde{v}}_{2}$. In general, ${\tilde{\alpha }}$ and ${\tilde{\beta }}$ can be viewed as control parameters that can be varied between experiments; all remaining parameters should remain constant between different experiments (at fixed temperatures and pHs), such assumptions being appropriate to the experimental approach we describe in Sect. 5. Hence, we are interested in inferring the three parameters with a bracketed superscript of 1, which are ${\tilde{k}}^{(1)}$, ${\tilde{K}}_1^{(1)}$, and ${\tilde{K}}_2^{(1)}$, from measurements of ${\tilde{s}}_6$.

It is apparent from the system (1)–(3), as well as from Fig. 2, that the concentrations for ${\tilde{s}}_3$, ${\tilde{s}}_6$, and ${\tilde{s}}_7$ decouple from the rest of the system. We include them here to keep the same notation as for Sect. 3. Moreover, we are able to reduce the system to two ODEs by noting the five following linearly independent conserved quantities:

4 $$\begin{aligned} {\tilde{s}}_1+ {\tilde{s}}_3= {\tilde{\alpha }}, \quad {\tilde{s}}_2+ {\tilde{s}}_3= {\tilde{\beta }}, \quad {\tilde{s}}_5+ {\tilde{s}}_6= {\tilde{\gamma }}, \quad {\tilde{s}}_2+ {\tilde{s}}_4+ {\tilde{s}}_6= {\tilde{\beta }}, \quad {\tilde{s}}_6= {\tilde{s}}_7. \end{aligned}$$

We delay the system reduction to discuss first the following standard assumptions made when carrying out a coupled enzyme assay, as this will aid in deciding with which ODEs to work. First, we assume that the maximum rate of reaction mediated by the auxiliary enzyme is much faster than for the primary enzyme, so that ${\tilde{k}}^{(2)}\gg {\tilde{k}}^{(1)}$. As the maximum rate of each reaction is proportional to the concentration of the corresponding enzyme, this can be ensured by using a high enough ratio of auxiliary to primary enzyme. Second, we will initially saturate the system with large quantities of ${\tilde{s}}_5$, so that ${\tilde{\gamma }}\gg {\tilde{K}}_5^{(2)}$, thus reducing the complexity of ${\tilde{v}}_{2}$ in (3). Third, we will initially saturate the system with either ${\tilde{s}}_1$ or ${\tilde{s}}_2$ (so that ${\tilde{\alpha }}\gg {\tilde{K}}_1^{(1)}$ or ${\tilde{\beta }}\gg {\tilde{K}}_2^{(1)}$) if we are interested in determining ${\tilde{K}}_2^{(1)}$ or ${\tilde{K}}_1^{(1)}$, respectively. This reduces the complexity of ${\tilde{v}}_{1}$ in (3). This final assumption is not required to facilitate a solution of the reduced system, though it does reduce the number of fitting parameters. In Sect. 3, we only make the first two assumptions in our analysis. Not making the third assumption allows us to consider the separate cases of saturating ${\tilde{s}}_1$ and ${\tilde{s}}_2$ at the same time.

We will present the method for determining ${\tilde{K}}_1^{(1)}$, essentially the case with a large initial saturation of ${\tilde{s}}_2$. The approach to determining ${\tilde{K}}_2^{(1)}$ is equivalent for the standard coupled enzyme assay discussed in this section, but this will not be the case for the non-standard assay we consider in Sect. 3. If we initially saturate the system with large quantities of ${\tilde{s}}_2$, it is convenient to use the following two ODEs to describe the system:

5 $$\begin{aligned} \dfrac{\mathrm {d} {\tilde{s}}_1}{\mathrm {d} {\tilde{t}}} = - \dfrac{{\tilde{k}}^{(1)}{\tilde{s}}_1}{{\tilde{K}}_1^{(1)}+ {\tilde{s}}_1}, \quad \dfrac{\mathrm {d} {\tilde{s}}_4}{\mathrm {d} {\tilde{t}}} = \dfrac{{\tilde{k}}^{(1)}{\tilde{s}}_1}{{\tilde{K}}_1^{(1)}+ {\tilde{s}}_1} - \dfrac{{\tilde{k}}^{(2)}{\tilde{s}}_4}{{\tilde{K}}_4^{(2)}+ {\tilde{s}}_4}, \quad {\tilde{s}}_1(0) = {\tilde{\alpha }}, \quad {\tilde{s}}_4(0) = 0. \end{aligned}$$

This is the standard system to consider when using coupled enzyme assays, investigated in the literature in McClure (1969), as discussed in Sect. 1.

To implement our systematic asymptotic analysis, we form dimensionless variables as in Table 1, and obtain the following system

6 $$\begin{aligned} \dfrac{\mathrm {d} s_1}{\mathrm {d} t} = - \epsilon a\dfrac{ s_1}{K_1^{(1)}+ s_1}, \quad \dfrac{\mathrm {d} s_4}{\mathrm {d} t} = \dfrac{ s_1}{K_1^{(1)}+ s_1} - \dfrac{ s_4}{1 + \epsilon s_4}, \quad s_1(0) = 1, \quad s_4(0) = 0, \end{aligned}$$

**Fig. 3:**
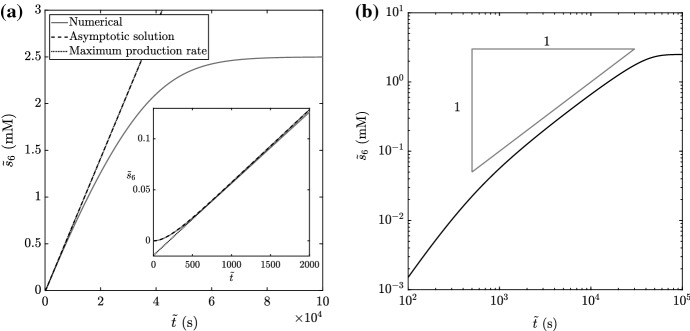
The measurable chemical ${\tilde{s}}_6$ as the numerical solution to (1), the asymptotic solution (8) (prior to the depletion dynamics), and the long-time asymptotic solution (9) (also prior to the depletion dynamics). We use the parameter values in Table 2, choosing ${\tilde{\alpha }}= 2.5$ mM and using ${\tilde{\beta }}= {\tilde{\gamma }}= 50$ mM to focus on the effect of a limiting initial concentration of ${\tilde{s}}_1$, rather than ${\tilde{s}}_2$ or ${\tilde{s}}_5$

where typical values and definitions of the dimensional and dimensionless parameters are given in Tables 2 and 3, respectively. The most important definition here is $\epsilon = {\tilde{k}}^{(1)}/{\tilde{k}}^{(2)}$ which, as discussed above, is small in coupled enzyme assays. We also define $a= {\tilde{K}}_4^{(2)}/{\tilde{\alpha }}$ and $K_1^{(1)}= {\tilde{K}}_1^{(1)}/{\tilde{\alpha }}$.

In the limit of $\epsilon \rightarrow 0$, with $t = \textit{O}(1)$, (6) has leading-order solution

7 $$\begin{aligned} s_1= 1, \quad s_4= \dfrac{1 - e^{-t}}{K_1^{(1)}+ 1}, \end{aligned}$$

which corresponds to the dimensional solution

8 $$\begin{aligned} {\tilde{s}}_6= \dfrac{{\tilde{\alpha }}{\tilde{k}}^{(1)}}{{\tilde{K}}_1^{(1)}+ {\tilde{\alpha }}} \left( {\tilde{t}}+ \dfrac{{\tilde{K}}_4^{(2)}}{{\tilde{k}}^{(2)}} \left[ \exp \left\{ -{\tilde{k}}^{(2)}{\tilde{t}}/ {\tilde{K}}_4^{(2)}\right\} - 1\right] \right) , \end{aligned}$$

for our measurable chemical. The long-time limit of (8) is

9 $$\begin{aligned} {\tilde{s}}_6\sim \dfrac{{\tilde{\alpha }}{\tilde{k}}^{(1)}}{{\tilde{K}}_1^{(1)}+ {\tilde{\alpha }}}\left( {\tilde{t}}- \dfrac{{\tilde{K}}_4^{(2)}}{{\tilde{k}}^{(2)}} \right) \quad \text {as } {\tilde{t}}\rightarrow \infty . \end{aligned}$$

Therefore, the kinetic parameters ${\tilde{k}}^{(1)}$ and ${\tilde{K}}_1^{(1)}$ can be inferred by measuring the long-term (by which we mean ${\tilde{K}}_4^{(2)}/{\tilde{k}}^{(2)}\ll {\tilde{t}}\ll {\tilde{\alpha }}/{\tilde{k}}^{(1)}$, for reasons noted below) production rate of ${\tilde{s}}_6$ for different values of ${\tilde{\alpha }}$. A similar procedure, but with saturating ${\tilde{s}}_1$ instead of ${\tilde{s}}_2$, allows the inference of ${\tilde{k}}^{(1)}$ (again) and ${\tilde{K}}_2^{(1)}$. The solution (9) tells us that the long-term production rate will be ${\tilde{\alpha }}{\tilde{k}}^{(1)}/({\tilde{K}}_1^{(1)}+ {\tilde{\alpha }})$, as can be seen in Fig. 3, and that we require ${\tilde{t}}\gg {\tilde{K}}_4^{(2)}/{\tilde{k}}^{(2)}$ before ${\tilde{s}}_6$ production will appear to be at a constant rate (${\tilde{K}}_4^{(2)}/{\tilde{k}}^{(2)}\approx 200$ s in Fig. 3). However, this constant rate will not occur indefinitely - given that $\mathrm {d}{s_1}/\mathrm {d}t = \textit{O}(a\epsilon )$, the production rate of ${\tilde{s}}_6$ will start to deviate from constant when $t = \textit{O}(1/ a\epsilon )$, equivalent to ${\tilde{t}}= \textit{O}({\tilde{\alpha }}/{\tilde{k}}^{(1)}) = \textit{O}(2.5 \times 10^4 \, s)$ in Fig. 3. In the language of matched asymptotic expansions, this corresponds to an outer scaling, a timescale over which (what we term) the depletion dynamics occur. For brevity, we do not consider the depletion dynamics further for the standard enzyme assay, but we will for the non-standard enzyme assay.

## Non-standard coupled enzyme assay

We now consider the main problem we are concerned with in this paper - the determination of the kinetic parameters ${\tilde{k}}^{(1)}$, ${\tilde{K}}_1^{(1)}$, and ${\tilde{K}}_2^{(1)}$ of the primary enzyme in the system shown in Fig. 1, where we assume knowledge of the kinetic parameters of the auxiliary enzyme. Experimentally, we are able to measure the concentration of NADH (*i.e.* ${\tilde{s}}_6$) in the system, and so our overarching goal is to understand how to use this measurement to infer the kinetic parameters that characterize the primary enzyme for a range of different substrates. The differences between the standard coupled enzyme assay in Sect. 2 and the problem we consider in this section are that the latter involves a reversible auxiliary reaction and that ${\tilde{s}}_1$ is a product/substrate of the forward/backward auxiliary reaction, rather than the auxiliary reaction being unidirectional and ${\tilde{s}}_1$ being decoupled from the auxiliary reaction in Sect. 2. The reaction network we consider is particularly relevant to the class of enzymes known as aminotransferases,^1^ as discussed in the Introduction.

The governing equations for the network shown in Fig. 1 consist of the following seven ODEs

10 $$\begin{aligned} \dfrac{\mathrm {d} {\tilde{s}}_1}{\mathrm {d} {\tilde{t}}}&= - {\tilde{v}}_{1}+ {\tilde{v}}_{2}- {\tilde{v}}_{-2}, \quad \dfrac{\mathrm {d} {\tilde{s}}_2}{\mathrm {d} {\tilde{t}}} = - {\tilde{v}}_{1}, \quad \dfrac{\mathrm {d} {\tilde{s}}_3}{\mathrm {d} {\tilde{t}}} = {\tilde{v}}_{1}, \nonumber \\ \dfrac{\mathrm {d} {\tilde{s}}_4}{\mathrm {d} {\tilde{t}}}&= {\tilde{v}}_{1}- {\tilde{v}}_{2}+ {\tilde{v}}_{-2}, \quad \dfrac{\mathrm {d} {\tilde{s}}_5}{\mathrm {d} {\tilde{t}}} = - {\tilde{v}}_{2}+ {\tilde{v}}_{-2}, \quad \dfrac{\mathrm {d} {\tilde{s}}_6}{\mathrm {d} {\tilde{t}}} = {\tilde{v}}_{2}- {\tilde{v}}_{-2}, \quad \dfrac{\mathrm {d} {\tilde{s}}_7}{\mathrm {d} {\tilde{t}}} = {\tilde{v}}_{2}- {\tilde{v}}_{-2}, \end{aligned}$$

where we define each variable in Table 1. As in the previous section, we use the convention that dimensional quantities are marked with a tilde, whereas dimensionless quantities are not. Here, the three reaction velocities are

11a $$\begin{aligned} {\tilde{v}}_{1}&= {\tilde{k}}^{(1)}\left( \dfrac{{\tilde{s}}_1}{{\tilde{K}}_1^{(1)}+ {\tilde{s}}_1}\right) \left( \dfrac{{\tilde{s}}_2}{{\tilde{K}}_2^{(1)}+ {\tilde{s}}_2}\right) , \end{aligned}$$

11b $$\begin{aligned} {\tilde{v}}_{2}&= {\tilde{k}}^{(2)}\left( \dfrac{{\tilde{s}}_4}{{\tilde{K}}_4^{(2)}+ {\tilde{s}}_4}\right) \left( \dfrac{{\tilde{s}}_5}{{\tilde{K}}_5^{(2)}+ {\tilde{s}}_5}\right) , \end{aligned}$$

11c $$\begin{aligned} {\tilde{v}}_{-2}&= {\tilde{k}}^{(-2)}\left( \dfrac{{\tilde{s}}_1}{{\tilde{K}}_{1}^{(-2)}+ {\tilde{s}}_1}\right) \left( \dfrac{{\tilde{s}}_6}{{\tilde{K}}_{6}^{(-2)}+ {\tilde{s}}_6}\right) \left( \dfrac{{\tilde{s}}_7}{{\tilde{K}}_{7}^{(-2)}+ {\tilde{s}}_7}\right) , \end{aligned}$$

each following Michaelis–Menten kinetics generalized to multiple substrates (Alberty 1953), under the assumption that the affinity a given substrate has for an enzyme is independent of the other substrates.

**Table 1:** Definitions of the dimensional and dimensionless variables in our system

Original variable	Description	Nondimensionalisation
${\tilde{s}}_1$	Pyruvate	${\tilde{s}}_1= {\tilde{\alpha }}s_1$
${\tilde{s}}_2$	Substrate	${\tilde{s}}_2= {\tilde{\beta }}s_2$
${\tilde{s}}_3$	Aldehyde	${\tilde{s}}_3= {\tilde{\beta }}s_3$
${\tilde{s}}_4$	Alpha-L-alanine	${\tilde{s}}_4= ({\tilde{k}}^{(1)}{\tilde{K}}_4^{(2)}/{\tilde{k}}^{(2)}) s_4$
${\tilde{s}}_5$	$\hbox {NAD}^{+}$	${\tilde{s}}_5= {\tilde{\gamma }}s_5$
${\tilde{s}}_6$	NADH	${\tilde{s}}_6= ({\tilde{k}}^{(1)}{\tilde{K}}_4^{(2)}/{\tilde{k}}^{(2)}) s_6$
${\tilde{t}}$	Time	${\tilde{t}}= ({\tilde{K}}_4^{(2)}/{\tilde{k}}^{(2)})t$

**Table 2:** Dimensional parameters and their typical size if known

Typical parameter size	Notes
${\tilde{k}}^{(1)}\approx 0.1 \, \upmu \mathrm {M s}^{-1}$	To be determined
${\tilde{K}}_1^{(1)}\approx 1 \, \mathrm {mM}$	To be determined
${\tilde{K}}_2^{(1)}\approx 1 \, \mathrm {mM}$	To be determined
${\tilde{k}}^{(2)}= 10 \, \upmu \mathrm {M s}^{-1}$	Experimental choice
${\tilde{K}}_4^{(2)}= 2 \, \mathrm {mM}$	From *Bacillus subtilis* (Yoshida and Freese 1965), though 0.45–$14 \, \mathrm {mM}$ from other organisms (Tolxdorff-Neutzling and Klemme 1982; Chowdhury et al. 1998; Sawa et al. 1994; Hutter and Singh 1999)
${\tilde{K}}_5^{(2)}= 0.2 \, \mathrm {mM}$	From *Bacillus subtilis* (Yoshida and Freese 1965), though 0.04–$0.3 \, \mathrm {mM}$ from other organisms (Tolxdorff-Neutzling and Klemme 1982; Chowdhury et al. 1998; Sawa et al. 1994; Hutter and Singh 1999)
${\tilde{k}}^{(-2)}= 0$–$2 \, \mathrm {mM s}^{-1}$	Value determined by choice of ${\tilde{k}}^{(2)}$ and assay environment.
${\tilde{K}}_{1}^{(-2)}= 0.5 \, \mathrm {mM}$	From *Bacillus subtilis* (Yoshida and Freese 1965), though 0.22–$1.45 \, \mathrm {mM}$ from other organisms (Tolxdorff-Neutzling and Klemme 1982; Chowdhury et al. 1998; Sawa et al. 1994; Hutter and Singh 1999)
${\tilde{K}}_{6}^{(-2)}= 0.02 \, \mathrm {mM}$	From *Bacillus subtilis* (Yoshida and Freese 1965), though 0.02–$0.1 \, \mathrm {mM}$ from other organisms (Tolxdorff-Neutzling and Klemme 1982; Chowdhury et al. 1998; Sawa et al. 1994; Hutter and Singh 1999)
${\tilde{K}}_{7}^{(-2)}= 40 \, \mathrm {mM}$	From *Bacillus subtilis* (Yoshida and Freese 1965), though 28–$67 \, \mathrm {mM}$ from other organisms (Tolxdorff-Neutzling and Klemme 1982; Chowdhury et al. 1998; Sawa et al. 1994; Hutter and Singh 1999)
${\tilde{\alpha }}= 1$–$5 \, \mathrm {mM}$	Control parameter
${\tilde{\beta }}= 1$–$5 \, \mathrm {mM}$	Control parameter
${\tilde{\gamma }}= 5 \, \mathrm {mM}$	Experimental choice

For the auxiliary enzyme, we use values taken from Yoshida and Freese (1965) in our numerical simulations. We give an idea of the range of these parameter values by also providing data from four other organisms: *Enterobacter aerogenes* (Chowdhury et al. 1998), *Mycobacterium tuberculosis* (Hutter and Singh 1999), *Phormidium lapideum* (Sawa et al. 1994), and *Rhodopseudomonas capsulata* (Hutter and Singh 1999)

In conjunction with the governing equations (10), we use initial conditions

12 $$\begin{aligned}&{\tilde{s}}_1(0) = {\tilde{\alpha }}, \quad {\tilde{s}}_2(0) = {\tilde{\beta }}, \quad {\tilde{s}}_3(0) = 0, \quad {\tilde{s}}_4(0) = 0, \nonumber \\&\quad {\tilde{s}}_5(0) = {\tilde{\gamma }}, \quad {\tilde{s}}_6(0) = 0, {\tilde{s}}_7(0) = 0, \end{aligned}$$

where ${\tilde{\alpha }}, {\tilde{\beta }}, {\tilde{\gamma }}> 0$. This corresponds to a system initially containing pyruvate (${\tilde{s}}_1$), an omega-amino acid substrate (${\tilde{s}}_2$), and $\hbox {NAD}^{+}$ (${\tilde{s}}_5$), with no other substrates present in the reaction network, then instantaneously adding the primary and auxiliary enzyme such that the entire system is well-mixed. Hence, there are thirteen dimensional parameters. We reiterate that our goal is to infer the three parameters ${\tilde{k}}^{(1)}$, ${\tilde{K}}_1^{(1)}$, and ${\tilde{K}}_2^{(1)}$ that correspond to the primary enzyme, assuming that we know the remaining ten parameters, which correspond to the auxiliary enzyme and the initial chemical concentrations. We provide typical parameter values in Table 2.

**Fig. 4:**
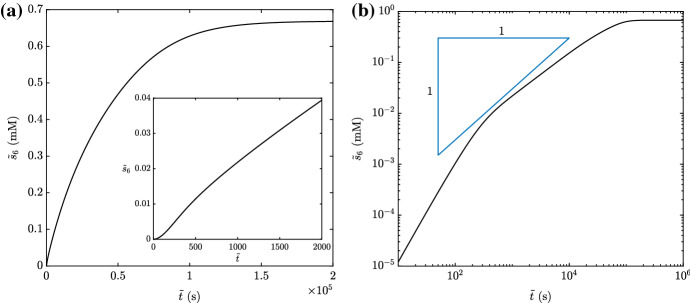
The measurable chemical ${\tilde{s}}_6$ as the numerical solution to (10) in **a** a linear plot and **b** a log-log plot. We use the parameter values in Table 2, with ${\tilde{k}}^{(-2)}= 0.5$ mM $\hbox {s}^{-1}$, ${\tilde{\alpha }}= 2.5$ mM, and ${\tilde{\beta }}= 2.5$ mM

As would be expected on physical grounds, the inclusion of a reverse reaction for the auxiliary enzyme causes less NADH to be made over a given time (Fig. 4a). Moreover, the reverse reaction can result in a linear phase that is very short (Fig. 4b), making the standard approach of calculating a steady production rate difficult to implement in practice. To this end, we will characterize the different possible types of NADH production through a systematic asymptotic analysis—see Bender and Orszag 2013; Kevorkian and Cole 2013 for general descriptions of the approach.

Before we nondimensionalize the system, we note from (10) and (12) the five following linearly independent conserved quantities:

13 $$\begin{aligned} {\tilde{s}}_1+ {\tilde{s}}_4= {\tilde{\alpha }}, \quad {\tilde{s}}_2+ {\tilde{s}}_3= {\tilde{\beta }}, \quad {\tilde{s}}_5+ {\tilde{s}}_6= {\tilde{\gamma }}, \quad {\tilde{s}}_2+ {\tilde{s}}_4+ {\tilde{s}}_6= {\tilde{\beta }}, \quad {\tilde{s}}_6= {\tilde{s}}_7. \end{aligned}$$

Thus, we may immediately reduce the number of ODEs from seven to two. However, the asymptotic analysis we will carry out is more intuitive if we keep most of the ODEs; the only ones we disregard are those for $\mathrm {d}{\tilde{s}}_3/\mathrm {d}{\tilde{t}}$ and $\mathrm {d}{\tilde{s}}_7/\mathrm {d}{\tilde{t}}$ in (10). For the former, we do this since the aldehyde produced from the primary enzyme (${\tilde{s}}_3$) decouples from the rest of the system, so we are able ignore this ODE henceforth, noting that ${\tilde{s}}_3$ can be determined from (13). For the latter, the concentration of ammonia is always equivalent to the concentration of NADH (${\tilde{s}}_7\equiv {\tilde{s}}_6$), so we will replace ${\tilde{s}}_7$ by ${\tilde{s}}_6$ henceforth.

We form the following dimensionless variables

14 $$\begin{aligned} {\tilde{t}}=&({\tilde{K}}_4^{(2)}/{\tilde{k}}^{(2)})t, \quad {\tilde{s}}_1= {\tilde{\alpha }}s_1, \quad {\tilde{s}}_2= {\tilde{\beta }}s_2, \nonumber \\&({\tilde{s}}_4, {\tilde{s}}_6) = ({\tilde{k}}^{(1)}{\tilde{K}}_4^{(2)}/{\tilde{k}}^{(2)}) (s_4,s_6), {\tilde{s}}_5= {\tilde{\gamma }}s_5, \end{aligned}$$

which we also summarize in Table 2. The timescale here (*i.e.* ${\tilde{K}}_4^{(2)}/{\tilde{k}}^{(2)}$) is that of initial alpha-L-alanine ($s_4$) and NADH ($s_6$) formation, and is 200 s for the parameters in Table 2. For the substrate dimensionless variables we scale pyruvate, beta-alanine, and $\hbox {NAD}^{+}$ with their initial conditions and alpha-L-alanine and NADH with the order of magnitude of alpha-L-alanine present during the regime of linear NADH growth, which our analysis will show to be approximately $50 \, \upmu$M. From (14), we obtain the dimensionless governing equations

15a $$\begin{aligned} \dfrac{\mathrm {d} s_1}{\mathrm {d} t}&= -\epsilon a\left( v_{1}- v_{2}+ v_{-2}\right) , \end{aligned}$$

15b $$\begin{aligned} \dfrac{\mathrm {d} s_2}{\mathrm {d} t}&= - \epsilon bv_{1}, \end{aligned}$$

15c $$\begin{aligned} \dfrac{\mathrm {d} s_4}{\mathrm {d} t}&= v_{1}- v_{2}+ v_{-2}, \end{aligned}$$

15d $$\begin{aligned} \dfrac{\mathrm {d} s_5}{\mathrm {d} t}&= - \epsilon c\left( v_{2}- v_{-2}\right) , \end{aligned}$$

15e $$\begin{aligned} \dfrac{\mathrm {d} s_6}{\mathrm {d} t}&= v_{2}- v_{-2}, \end{aligned}$$

where the three dimensionless reaction velocities are defined as

16a $$\begin{aligned} v_{1}&= \left( \dfrac{s_1}{K_1^{(1)}+ s_1}\right) \left( \dfrac{s_2}{K_2^{(1)}+ s_2}\right) , \end{aligned}$$

16b $$\begin{aligned} v_{2}&= \left( \dfrac{s_4}{1 + \epsilon s_4}\right) \left( \dfrac{s_5}{\delta + s_5}\right) , \end{aligned}$$

16c $$\begin{aligned} v_{-2}&= k^{(-2)}K\left( \dfrac{s_1}{K_{1}^{(-2)}+ s_1}\right) \left( \dfrac{s_6}{K_{6}^{(-2)}+ s_6}\right) \left( \dfrac{s_6}{1 + \epsilon Ks_6}\right) . \end{aligned}$$

The initial conditions of the dimensionless system are

17 $$\begin{aligned} s_1(0) = 1, \quad s_2(0) = 1, \quad s_4(0) = 0, \quad s_5(0) = 1, \quad s_6(0) = 0. \end{aligned}$$

We provide the definitions of the dimensionless parameters in Table 3, from which we note that $\epsilon$, $\delta$, and $K$ are all much smaller than 1. Additionally, we note that the value of $k^{(-2)}$, representing the ratio of the backward to forward rates controlled by the auxiliary enzyme, can vary across many orders of magnitude.

We reiterate that our goal is to understand how to relate the measurement of NADH (${\tilde{s}}_6$) in the linear production regime to the values of the kinetic parameters governing the primary enzyme. Our approach is to comprehensively investigate how the system behaves for different magnitudes of $k^{(-2)}$, with a focus on understanding when a linear regime for NADH production is appropriate and what the measurement of NADH in this regime will tell us about the primary enzyme. To this end, we perform an asymptotic analysis that exploits the small parameters $\epsilon$, $\delta$, and $K$ (noting that the three limits all commute), with a focus on analysing how the asymptotic size of $k^{(-2)}$ affects the system behaviour. We emphasize that the parameter $\epsilon$ can always be made to be small by suitable choices of the relative concentrations of the primary and auxiliary enzyme, which scale with ${\tilde{k}}^{(1)}$ and ${\tilde{k}}^{(2)}$, respectively, and likewise for $\delta$ by choosing ${\tilde{\gamma }}$, the initial concentration of ${\tilde{s}}_5$, to be significantly larger than ${\tilde{K}}_5^{(2)}$, the Michaelis constant for ${\tilde{s}}_5$ in the forward auxiliary reaction. Since the smallness of these parameters reduces the complexity of the system, it is experimentally favourable to choose the relative enzyme concentrations and initial conditions to make $\epsilon$ and $\delta$ small. We note that the smallness of $K$ is slightly different, however. While the typical parameter values we give in Table 2 unanimously result in small values of $K$, there seems no fundamental reason why it should be small. To ensure the generality of our analysis, we therefore also present results for $K= \textit{O}(1)$ in “Appendix C”.

It turns out that there are two distinguished asymptotic limits in this system (so that all other regimes with $\epsilon$, $\delta$, and $K$ each small are sub-cases of these two), whereby $k^{(-2)}= \textit{O}(1/K) \gg 1$ and $k^{(-2)}= \textit{O}(\epsilon K) \ll 1$, respectively. We investigate the former in the next section, discussing its relevance to our inference problem, and we investigate the latter in Sect. 3.2, where we show that it can be considered a sub-limit of the former case in terms of experimental measurements. Moreover, we show that the latter regime exhibits the same constant ‘long-time’ production rate of NADH as the standard coupled enzyme assay case we summarized in Sect. 2. In Table 4, we provide a summary of the results we derive in the remainder of this section.

**Table 3:** The dimensionless parameters in our system, obtained using the data in Table 2

$\epsilon = \dfrac{{\tilde{k}}^{(1)}}{{\tilde{k}}^{(2)}} \approx 0.01$	$b= \dfrac{{\tilde{K}}_4^{(2)}}{{\tilde{\beta }}} = 0.4$ – 2	$K_{6}^{(-2)}= \dfrac{{\tilde{K}}_{6}^{(-2)}{\tilde{k}}^{(2)}}{{\tilde{K}}_4^{(2)}{\tilde{k}}^{(1)}} \approx 1$
$K_1^{(1)}= \dfrac{{\tilde{K}}_1^{(1)}}{{\tilde{\alpha }}} \approx 0.2$ – 1	$c= \dfrac{{\tilde{K}}_4^{(2)}}{{\tilde{\gamma }}} = 0.8$	$k^{(-2)}= \dfrac{{\tilde{k}}^{(-2)}}{{\tilde{k}}^{(2)}} = 0$ – $10^{3}$
$K_2^{(1)}= \dfrac{{\tilde{K}}_2^{(1)}}{{\tilde{\beta }}} \approx 0.2$ – 1	$\delta = \dfrac{{\tilde{K}}_5^{(2)}}{{\tilde{\gamma }}} = 0.04$	$K= \dfrac{{\tilde{K}}_4^{(2)}}{{\tilde{K}}_{7}^{(-2)}} = 0.05$
$a= \dfrac{{\tilde{K}}_4^{(2)}}{{\tilde{\alpha }}} = 0.4$ – 2	$K_{1}^{(-2)}= \dfrac{{\tilde{K}}_{1}^{(-2)}}{{\tilde{\alpha }}} \approx 0.1$ - 0.5	

The ‘approximately equals to’ signs arise from uncertainty in the kinetic parameters of the primary enzyme

**Table 4:**
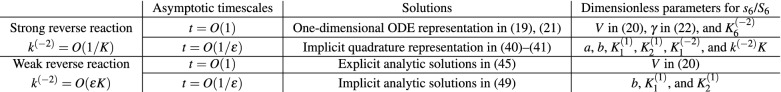
A summary of the solutions we derive in this paper for each timescale in the two distinguished limits of the problem, as well as the dimensionless parameters that control the concentration of $s_6$ in each asymptotic region

We give the equation number for the definition of each parameter unless it is given in Table 3

### Strong reverse reaction: $k^{(-2)}= \textit{O}(1/K)$

When $k^{(-2)}= \textit{O}(1/K)$, the auxiliary-enzyme-controlled reaction in the forward direction is much stronger than for the reverse direction. This scaling results in a distinguished limit where the nonlinearity of the reverse reaction is important when $t = \textit{O}(1)$, which is where the linear growth regime occurs for the standard coupled enzyme assay. We now exploit the limits $\epsilon , \delta , K\ll 1$ with $k^{(-2)}K= \textit{O}(1)$. We note that while small values of $\epsilon$ would result in large values of $K_{6}^{(-2)}$, we will treat $K_{6}^{(-2)}= \textit{O}(1)$ (as we do with the remaining dimensionless parameters in the system) as this choice keeps more terms at leading order (*i.e.* constitutes a distinguished limit) and is the case we expect from experimental parameters.

#### Linear-phase regime

We first consider the linear-phase regime, which occurs over the timescale $t = \textit{O}(1)$. By ‘linear-phase’, we mean that $s_6$ will exhibit linear growth for some time period which we can approximate. In the language of matched asymptotic expansions, this corresponds to linear growth in the intermediate matching region between this timescale and the longer depletion timescale (which we will show corresponds to $t = \textit{O}(1/\epsilon )$).

In the limits set out in Sect. 3.1, the leading-order version of (15) for $t = \textit{O}(1)$ is

18a $$\begin{aligned} \dfrac{\mathrm {d} s_1}{\mathrm {d} t}&= 0, \end{aligned}$$

18b $$\begin{aligned} \dfrac{\mathrm {d} s_2}{\mathrm {d} t}&= 0, \end{aligned}$$

18c $$\begin{aligned} \dfrac{\mathrm {d} s_4}{\mathrm {d} t}&{=} \left( \dfrac{s_1}{K_1^{(1)}{+} s_1}\right) \left( \dfrac{s_2}{K_2^{(1)}{+} s_2}\right) {-} s_4{+} k^{(-2)}K\left( \dfrac{s_1}{K_{1}^{(-2)}+ s_1}\right) \left( \dfrac{s_6}{K_{6}^{(-2)}+ s_6}\right) s_6, \end{aligned}$$

18d $$\begin{aligned} \dfrac{\mathrm {d} s_5}{\mathrm {d} t}&= 0, \end{aligned}$$

18e $$\begin{aligned} \dfrac{\mathrm {d} s_6}{\mathrm {d} t}&= s_4- k^{(-2)}K\left( \dfrac{s_1}{K_{1}^{(-2)}+ s_1}\right) \left( \dfrac{s_6}{K_{6}^{(-2)}+ s_6}\right) s_6, \end{aligned}$$

with initial conditions (17). We may immediately deduce that, over this timescale,

19 $$\begin{aligned} s_1= 1, \quad s_2= 1, \quad s_5= 1, \quad s_4+ s_6= V t, \end{aligned}$$

where we define

20 $$\begin{aligned} V := \dfrac{1}{\left( K_1^{(1)}+ 1 \right) \left( K_2^{(1)}+ 1 \right) }. \end{aligned}$$

We can therefore reduce the system (17)–(18) to the following single ODE

21 $$\begin{aligned} \dfrac{\mathrm {d} s_6}{\mathrm {d} t} = V t - s_6- \dfrac{\gamma s_6^2}{K_{6}^{(-2)}+ s_6}, \quad s_6(0) = 0, \end{aligned}$$

where we define

22 $$\begin{aligned} \gamma := \dfrac{k^{(-2)}K}{K_{1}^{(-2)}+ 1}. \end{aligned}$$

Although we cannot solve (21) explicitly, we may deduce from (21) that the early-time behaviour is

23 $$\begin{aligned} s_6\sim \dfrac{V t^2}{2} \quad \text {as } t \rightarrow 0^{+}. \end{aligned}$$

and the long-time behaviour is

24 $$\begin{aligned} s_6\sim \dfrac{V t}{1 + \gamma } + \dfrac{\gamma K_{6}^{(-2)}(1+\gamma ) - V}{\left( 1 + \gamma \right) ^2} \quad \text {as } t \rightarrow \infty . \end{aligned}$$

**Fig. 5:**
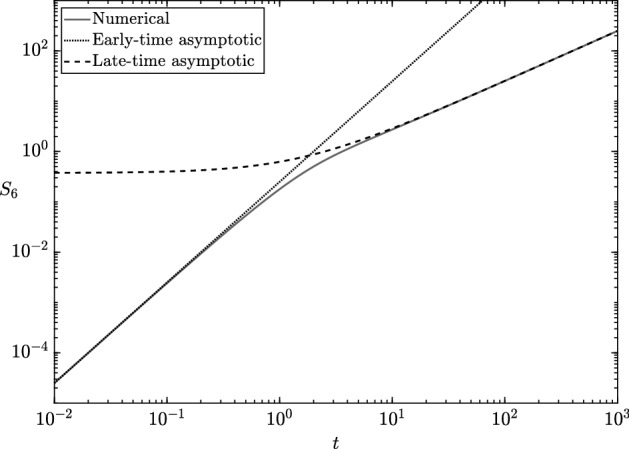
A comparison of the numerical solution to the ODE (21) with the early- and late-time asymptotic solutions (23) and (24), respectively. We use parameter values $V = 0.5$, $\gamma = 1$, $K_{6}^{(-2)}= 1$

These asymptotic solutions are good approximations of the numerical solution to (21) in their regions of validity (Fig. 5).

It will also be helpful to note that

25 $$\begin{aligned} s_4\sim \dfrac{\gamma V t}{1 + \gamma } \quad \text {as } t \rightarrow \infty . \end{aligned}$$

Hence when measuring $s_6$ experimentally, we will not observe linear growth in time immediately (rather, (23) implies that we will observe a $t^2$ relationship), but this linear relationship will start to develop after a lag period. To help understand further the solution of the ODE (21), we first note that we can reduce its dependence from three parameters to two by making the scaling $s_6= K_{6}^{(-2)}Y$. This results in the system

26a $$\begin{aligned} \dfrac{\mathrm {d} Y}{\mathrm {d} t} = {\bar{V}} t - Y - \dfrac{\gamma Y^2}{1 + Y}, \quad Y(0) = 0, \end{aligned}$$

where we define

26b $$\begin{aligned} {\bar{V}} = V/K_{6}^{(-2)}. \end{aligned}$$

**Fig. 6:**
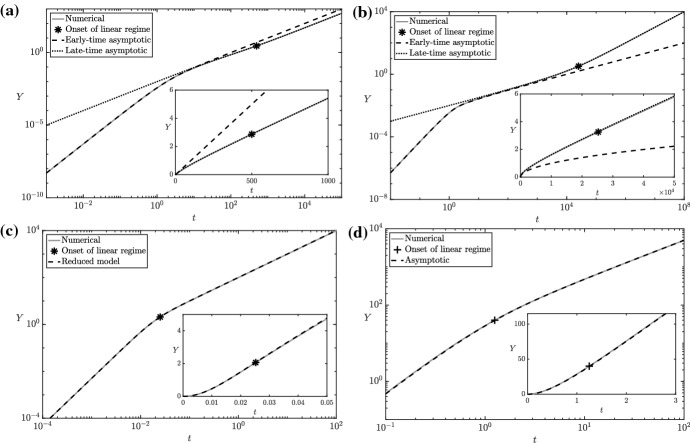
A comparison of numerical and asymptotic solutions of the reduced ODE (26) for **a** ${\bar{V}} = 10^{-2}$, $\gamma = 10^{0}$, **b** ${\bar{V}} = 10^{-2}$, $\gamma = 10^{2}$**c** ${\bar{V}} = 10^{4}$, $\gamma = 10^{2}$, **d** ${\bar{V}} = 10^{2}$, $\gamma = 10^{0}$. The onset of the linear regime is set as 2.5 times the right-hand side of (30), and is denoted by a cross/asterisk when the first/second term on the right-hand side is maximal

**Fig. 7:**
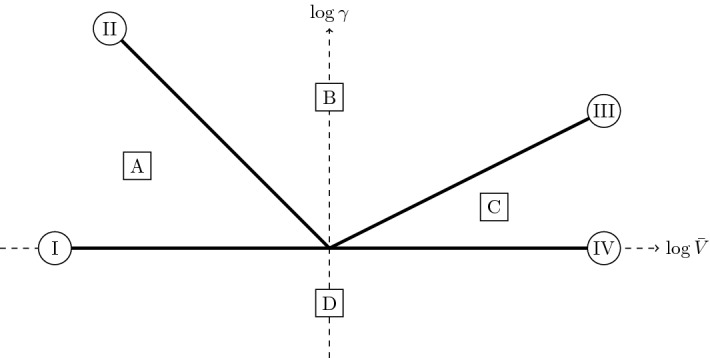
The distinguished asymptotic limits of the reduced ODE (26) for asymptotically large or small values of ${\bar{V}}$ and $\gamma$, denoted by dark solid lines and labelled using Roman numerals contained within a circle. Note that the distinguished limit I corresponds to ${\bar{V}} \ll 1$ and $\gamma = \textit{O}(1)$, the distinguished limit II corresponds to ${\bar{V}} \gamma = \textit{O}(1)$ with ${\bar{V}} \ll 1$ and $\gamma \gg 1$, the distinguished limit III corresponds to ${\bar{V}}/\gamma ^2 = \textit{O}(1)$ with ${\bar{V}} \gg 1$ and $\gamma \gg 1$, and the distinguished limit IV corresponds to ${\bar{V}} \gg 1$ and $\gamma = \textit{O}(1)$. The sublimits of the distinguished asymptotic limits are noted using letters contained within a square

Solutions to (26) generally exhibit a transient region before settling to linear growth for a wide range of parameter values (Fig. 6). The long-time linear growth exhibited by the ODE (26) is

27 $$\begin{aligned} Y \sim \dfrac{{\bar{V}} t}{1 + \gamma } \quad \text {as } t \rightarrow \infty , \end{aligned}$$

and it is of interest to understand when this linear growth becomes apparent. One way to do this is to examine the menagerie of distinguished asymptotic limits of the ODE (26), which are interesting in their own right. Moreover, the analytic solutions afforded by an asymptotic analysis can be used to fit time-series data from experimental results more easily than numerical solutions of a system of ODEs. We investigate the subsidiary distinguished asymptotic limits in terms of the two parameters ${\bar{V}}$ and $\gamma$ in “Appendix A”, and we show a comparison of numerical and asymptotic solutions to (26) in Fig. 6. We illustrate the four distinguished limits that we obtain from the analysis of “Appendix A” in Fig. 7. A result from this analysis with particular importance for accurately interpreting the data is that there are two distinct linear regimes in the distinguished limit I.

While the duration of this transient region can be calculated numerically for given parameter values or approximated analytically from the asymptotic results we derive in “Appendix A”, it is helpful to obtain an approximate analytic estimate for when we expect to see a linear relationship for the general case. To this end, we first note that $Y \geqslant 0$, ${\dot{Y}} \geqslant 0$, and $Y/(1 + Y) \in [0, 1)$, so we may obtain the bounds $f_1(t) \leqslant Y(t) \leqslant f_2(t)$ where $f_1$ and $f_2$ satisfy the following ODEs

28 $$\begin{aligned} \dfrac{\mathrm {d} f_1}{\mathrm {d} t} = {\bar{V}} t - (1 + \gamma )f_1, \quad \dfrac{\mathrm {d} f_2}{\mathrm {d} t} = {\bar{V}} t - f_2, \quad f_1(0) = f_2(0) = 0, \end{aligned}$$

which yield the following bounds

29 $$\begin{aligned} \dfrac{{\bar{V}}}{\left( 1 + \gamma \right) ^2} \left[ \left( 1 + \gamma \right) t - 1 + e^{-\left( 1 + \gamma \right) t}\right] \leqslant Y(t) < {\bar{V}} \left( t - 1 + e^{-t}\right) . \end{aligned}$$

We note that the long-time limit of the lower bound in (29) is the long-time limit (27) of the ODE (26) since the nonlinear term in the ODE becomes negligible when $Y \gg 1$. Mathematically, the lower bound of (29) appears linear when $(1 + \gamma )t \gg 1$. Combining these two constraints, we expect the NADH concentration to grow linearly in time when

30 $$\begin{aligned} t \gg \max \left( \dfrac{1}{1 + \gamma }, \dfrac{\left( 1 + \gamma \right) }{{\bar{V}}} \right) . \end{aligned}$$

As $1/(1 + \gamma )$ is bounded above by 1, it is the second of these constraints that can lead to very large times before the linear regime is encountered. This problem is exacerbated when $\gamma \gg 1$ and ${\bar{V}} = V/K_{6}^{(-2)}\ll 1$, as can be seen by the significantly large times until linearity for Fig. 6b. Moreover, we note that the two constraints in (30) approximately balance when ${\bar{V}} = \textit{O}(\gamma ^2)$ for large $\gamma$ (essentially Regime III in Fig. 7), and where ${\bar{V}} = \textit{O}(1)$ for small $\gamma$. This represents a boundary between the two different constraints, where the large-${\bar{V}}$ case (on the right of Fig. 7) corresponds to $t \gg 1/(1 + \gamma )$. When we denote the onset of the linear regime in Fig. 6, we use an asterisk when $(1 + \gamma )/{\bar{V}} > 1/(1 + \gamma )$ and a cross when $(1 + \gamma )/{\bar{V}} < 1/(1 + \gamma )$. In each case, we take for definiteness the onset to occur at 2.5 times the value of the right-hand side of (30).

#### Physical interpretation

To understand the physical implications of our analysis from the linear-phase regime, it is helpful to reframe our results in terms of dimensional quantities. Hence, the long-time dimensional linear behaviour of NADH, given in (24) for the dimensionless problem, is

31 $$\begin{aligned} {\tilde{s}}_6\sim {\tilde{V}}\omega {\tilde{t}}\quad \text {as } {\tilde{t}}\rightarrow \infty , \end{aligned}$$

at leading order, where

32a $$\begin{aligned} \omega&= \dfrac{1}{1 + \dfrac{{\tilde{k}}^{(-2)}{\tilde{K}}_4^{(2)}}{{\tilde{k}}^{(2)}{\tilde{K}}_{7}^{(-2)}} \dfrac{{\tilde{\alpha }}}{{\tilde{K}}_{1}^{(-2)}+ {\tilde{\alpha }}}} \in (0,1), \end{aligned}$$

32b $$\begin{aligned} {\tilde{V}}&= {\tilde{k}}^{(1)}\dfrac{{\tilde{\alpha }}}{{\tilde{K}}_1^{(1)}+ {\tilde{\alpha }}} \dfrac{{\tilde{\beta }}}{{\tilde{K}}_2^{(1)}+ {\tilde{\beta }}}. \end{aligned}$$

Here, $\omega$ can be thought of as a measure of the relative strengths of the forward and backward auxiliary reactions: $\omega \rightarrow 0^{+}$ when the backward reaction is much stronger, but $\omega \rightarrow 1^{-}$ when the forward reaction is much stronger. Additionally, ${\tilde{V}}$ can be thought of as the ‘natural’ observed strength of the reaction, as this is the reaction strength when the backward auxiliary reaction is unimportant, as we show in Sect. 3.2. Moreover, $\omega$ can be calculated from the experimental setup, so the standard practice of inferring the unknown parameters for a single enzyme can be used, as long as the observed reaction velocity is modified by a factor of $1/\omega$. Thus, we have determined the reaction velocity in the linear-phase regime in terms of the system parameters.

Our remaining task is to understand when we expect to observe this regime. The dimensional version of the lower constraint for the linear regime, given in (30) for the dimensionless problem, is

33 $$\begin{aligned} {\tilde{t}}\gg \max \left( \dfrac{\omega {\tilde{K}}_4^{(2)}}{{\tilde{k}}^{(2)}}, \dfrac{{\tilde{K}}_{6}^{(-2)}}{{\tilde{V}}\omega } \right) . \end{aligned}$$

Hence, this gives us the approximate time we expect to wait before observing a linear relationship between time and NADH production. The constraint (33) provides a warning against particularly low initial concentrations of the primary enzyme, pyruvate, or the substrate, as this will cause a very large lag time. One can use (33) to specify a required lag time, and determine constraints for the initial concentrations discussed above.

However, while we have identified and characterized the linear regime of NADH production, this regime is unfortunately transient; it can only last until substrate depletion starts to affect the system at leading order, which occurs when *t* becomes of $\textit{O}(1/\epsilon )$. We investigate this regime in the next subsection.

#### Depletion regime

To investigate the depletion regime, we introduce $T = \epsilon t$ and analyse the limit $\epsilon \rightarrow 0$ with $T = \textit{O}(1)$. Moreover, from (24) and (25), we see that we must also make the scalings $S_4= s_4/\epsilon$, and $S_6= s_6/\epsilon$, resulting in the system

34a $$\begin{aligned} \epsilon \dfrac{\mathrm {d} s_1}{\mathrm {d} T}&= - a\left( \epsilon v_{1}- V_{2}+ V_{-2}\right) , \end{aligned}$$

34b $$\begin{aligned} \dfrac{\mathrm {d} s_2}{\mathrm {d} T}&= - bv_{1}, \end{aligned}$$

34c $$\begin{aligned} \epsilon \dfrac{\mathrm {d} S_4}{\mathrm {d} T}&= \epsilon v_{1}-V_{2}+ V_{-2}, \end{aligned}$$

34d $$\begin{aligned} \epsilon \dfrac{\mathrm {d} s_5}{\mathrm {d} T}&= - c\left( V_{2}- V_{-2}\right) , \end{aligned}$$

34e $$\begin{aligned} \epsilon \dfrac{\mathrm {d} S_6}{\mathrm {d} T}&= V_{2}- V_{-2}, \end{aligned}$$

where the three reaction velocities are now defined as

35a $$\begin{aligned} v_{1}&= \left( \dfrac{s_1}{K_1^{(1)}+ s_1}\right) \left( \dfrac{s_2}{K_2^{(1)}+ s_2}\right) , \end{aligned}$$

35b $$\begin{aligned} V_{2}&= \left( \dfrac{S_4}{1 + S_4}\right) \left( \dfrac{s_5}{\delta + s_5}\right) , \end{aligned}$$

35c $$\begin{aligned} V_{-2}&= k^{(-2)}K\left( \dfrac{s_1}{K_{1}^{(-2)}+ s_1}\right) \left( \dfrac{S_6}{\epsilon K_{6}^{(-2)}+ S_6}\right) \left( \dfrac{S_6}{1 + KS_6}\right) . \end{aligned}$$

The matching conditions from the earlier timescale yield the following ‘initial conditions’

36 $$\begin{aligned} s_1(0) = 1, \quad s_2(0) = 1, \quad S_4(0) = 0, \quad s_5(0) = 1, \quad S_6(0) = 0. \end{aligned}$$

Naively taking the limits of $\epsilon , \delta , K\rightarrow 0$ in (34) to obtain a leading-order system would yield a duplication of information. To avoid this, we must form appropriate linear combinations of the governing equations in order to obtain enough information for a leading-order system. Using this approach, exploiting conserved quantities where possible, we obtain the following differential-algebraic system at leading-order

37a $$\begin{aligned} \dfrac{\mathrm {d} s_2}{\mathrm {d} T}&= - b\left( \dfrac{s_1}{K_1^{(1)}+ s_1}\right) \left( \dfrac{s_2}{K_2^{(1)}+ s_2}\right) , \end{aligned}$$

37b $$\begin{aligned} \dfrac{S_4}{1 + S_4}&= k^{(-2)}K\left( \dfrac{s_1}{K_{1}^{(-2)}+ s_1}\right) S_6, \end{aligned}$$

37c $$\begin{aligned} s_1+ aS_4&= 1, \end{aligned}$$

37d $$\begin{aligned} s_2+ bS_4+ bS_6&= 1, \end{aligned}$$

37e $$\begin{aligned} s_5+ cS_6&= 1. \end{aligned}$$

Thus, we have reduced the problem to that of one ODE with four additional algebraic relationships, and the initial conditions (36). To further simplify this system, it is convenient to use (37b–d) to write

38 $$\begin{aligned} s_2= f(s_1) := 1 - \dfrac{b}{a} \left[ 1 - s_1+ \dfrac{a}{k^{(-2)}K} \left( \dfrac{s_1+ K_{1}^{(-2)}}{s_1}\right) \left( \dfrac{1 - s_1}{a+ 1 - s_1}\right) \right] , \end{aligned}$$

then to transform (37a) into a separable ODE for $s_1$, obtaining

39 $$\begin{aligned} \dfrac{\mathrm {d} s_1}{\mathrm {d} T}&= - \dfrac{ b}{f'(s_1)} \left( \dfrac{s_1}{K_1^{(1)}+ s_1}\right) \left( \dfrac{f(s_1)}{K_2^{(1)}+ f(s_1)}\right) , \quad s_1(0) = 1. \end{aligned}$$

Solving (39) allows the remaining variables to be deduced algebraically from (37b–e), (38). We are able to integrate (39) to obtain the following implicit solution

40a $$\begin{aligned} - bT&= f(s_1) - 1 + K_2^{(1)}\log f(s_1) + bK_1^{(1)}\left( \dfrac{\log s_1}{a} + \dfrac{g(s_1)}{k^{(-2)}K(a+1)^2} \right) \nonumber \\&\quad + K_1^{(1)}K_2^{(1)}\int _1^{s_1} \! \dfrac{f'(u)}{u f(u)} \, \mathrm {d}u, \end{aligned}$$

where

40b $$\begin{aligned} g(s_1)&: = \dfrac{K_{1}^{(-2)}\left( a+ 1 \right) }{2} \dfrac{s_1^2 - 1}{s_1^2} \nonumber \\&\quad + \dfrac{a(a+ 1 + K_{1}^{(-2)})}{a+1} \log \dfrac{as_1}{a+ 1 - s_1} - (a+ 1 + K_{1}^{(-2)}) \dfrac{1 - s_1}{a+ 1 - s_1}. \end{aligned}$$

The term with coefficient $bK_1^{(1)}$ on the right-hand side of (40a) arises from direct term-by-term integration of the ratio of the derivative of $f(s_1)$ (defined in (38)) and $s_1$.

Thus, we have characterized the depletion regime. One can use (37c–d), and (40) to obtain the following functional forms for the chemical concentrations in the depletion regime

41 $$\begin{aligned} s_2&= f(s_1), \quad S_4= \dfrac{1 - s_1}{a}, \quad s_5= 1 - c\left( \dfrac{1 - f(s_1)}{b} - \dfrac{1 - s_1}{a}\right) , \quad \nonumber \\ S_6&= \dfrac{1 - f(s_1)}{b} - \dfrac{1 - s_1}{a}. \end{aligned}$$

**Fig. 8:**
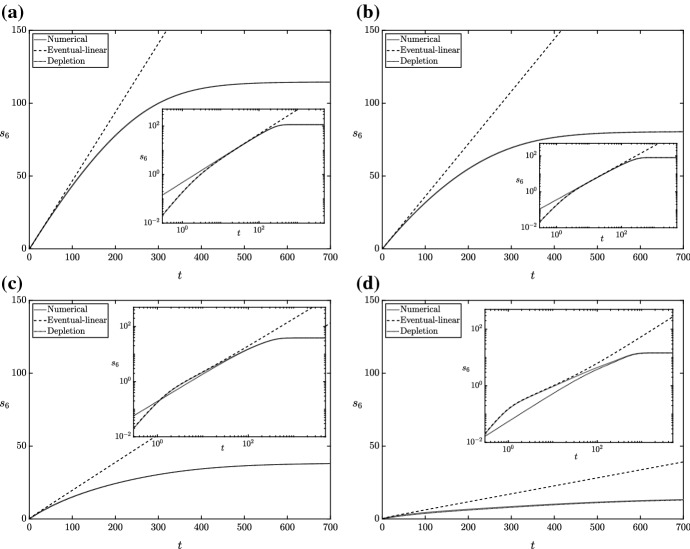
A comparison of numerical solutions of the full system (15) to the asymptotic or reduced solutions in the linear-phase regime from the previous section and in the depletion regime (40)–(41). We use the parameter values in Tables 2 and 3, with ${\tilde{\alpha }}= {\tilde{\beta }}= 2.5$ mM, and varying $k^{(-2)}$ in each sub-figure such that **a** $k^{(-2)}K= 0.1$, **b** $k^{(-2)}K= 0.5$, **c** $k^{(-2)}K= 2$, and **d** $k^{(-2)}K= 10$

The asymptotic solution for $S_6$ in (41) agrees well with the numerical results for a range of values of $k^{(-2)}$ in both the linear-phase regime of the previous section and the depletion regime of this section (Fig. 8).

While the transient-linear regime of the previous section is much easier to fit and has the benefit of being easier to identify from time data, this linear behaviour may only be exhibited for a short duration. Given experimental data on the NADH concentration, we can therefore estimate the three unknown parameters $k^{(1)}$, $K_1^{(1)}$, and $K_2^{(1)}$ via a nonlinear parameter fitting of (41). In practice, it is likely that either $s_1$ or $s_2$ will be saturating, corresponding to either $K_1^{(1)}$ or $K_2^{(1)}$ being small, thus reducing the number of parameters that need to be fitted at any one time. It is worth noting that, unlike in the standard assay considered in Sect. 2, the form of $S_6$ is not symmetric in the parameters $K_1^{(1)}$ and $K_2^{(1)}$. This is to be expected, since the symmetry in the reaction network is broken in the non-standard assay.

To identify when we may be able to use the transient-linear regime to fit data, we now estimate an upper bound for this regime. While it is possible to determine this by obtaining an early-time solution to the system (37) to calculate a correction to the long-time solution from the earlier time regime (24), the resultant expression is cumbersome and not particularly insightful. Instead, we use the following more crude approximation for an upper limit to the linear regime:

42 $$\begin{aligned} t \ll 1/a\epsilon , \end{aligned}$$

obtained from the requirement to stray from linearity in this depletion regime. In terms of dimensional quantities, (42) is

43 $$\begin{aligned} {\tilde{t}}\ll \dfrac{{\tilde{\alpha }}}{{\tilde{k}}^{(1)}}. \end{aligned}$$

Over the timescale $t = \textit{O}(1/a\epsilon )$, the levels of $S_6$ increase and the levels of $s_2$ and $s_5$ decrease. From (41), we see that the maximum amount of NADH produced is bounded above by an $\textit{O}(1)$ quantity. Hence, we see from (34)–(35) that there will be additional depletion dynamics if $s_5= \textit{O}(\delta )$. This condition is not guaranteed to occur, and will depend on the values of the kinetic parameters, as well as on the initial conditions of the system. We do not consider this depletion possibility further, as it has no bearing on our main goal of characterizing the primary enzyme. We consider the remaining distinguished limit in the system, that of a weak reverse reaction, in the next section.

### Weak reverse reaction: $k^{(-2)}= \textit{O}(\epsilon K)$

#### Linear-phase regime

The distinguished limit where the reverse reaction is weak occurs when $k^{(-2)}= \textit{O}(\epsilon K)$, and it will be helpful to introduce $\rho := k^{(-2)}/(\epsilon K) = \textit{O}(1)$ for notational purposes. This scaling results in a distinguished limit in which the nonlinearities of the primary reaction and the reverse auxiliary reaction are important when $t = \textit{O}(1/\epsilon )$. This causes the limit to be distinguished for $s_4$ over a timescale of $t = \textit{O}(1/\epsilon )$.

In this case, the leading-order version of (15) for $t = \textit{O}(1)$ is

44 $$\begin{aligned} \dfrac{\mathrm {d} s_1}{\mathrm {d} t} {=} 0, \quad \dfrac{\mathrm {d} s_2}{\mathrm {d} t} {=} 0, \quad \dfrac{\mathrm {d} s_4}{\mathrm {d} t} = \left( \dfrac{s_1}{K_1^{(1)}+ s_1}\right) \left( \dfrac{s_2}{K_2^{(1)}+ s_2}\right) - s_4, \quad \dfrac{\mathrm {d} s_5}{\mathrm {d} t} = 0, \dfrac{\mathrm {d} s_6}{\mathrm {d} t} {=} s_4, \end{aligned}$$

with initial conditions (17). The solution to (44) is

45 $$\begin{aligned} s_1= 1, \quad s_2= 1, \quad s_4= V \left( 1 - e^{-t} \right) , \quad s_5= 1, \quad s_6= V \left( t - 1 + e^{-t} \right) , \end{aligned}$$

where *V* is defined in (20). Hence, $s_6$ initially scales with $t^2$ before eventually scaling with *t*. Thus, when $t = \textit{O}(1)$ for a weak reverse reaction, the observed NADH production is essentially equivalent to that of Sect. 2, even though pyruvate is both a substrate of the primary reaction and a product of the auxiliary reaction. This is because there is not enough alpha-L-alanine being created to produce a large enough amount of pyruvate to affect the NADH production, and the reverse reaction is too weak to siphon away the NADH that is produced by a significant amount. In dimensional terms, the observed long-time concentration of NADH in this regime is

46 $$\begin{aligned} {\tilde{s}}_6\sim {\tilde{V}}{\tilde{t}}\quad \text {as } {\tilde{t}}\rightarrow \infty , \end{aligned}$$

essentially the sub-limit of (31) as $\omega \rightarrow 1$, with ${\tilde{V}}$ and $\omega$ defined in (32). Additionally, we are able to obtain a lower bound for this linear regime from (45), namely that $t \gg 1$ for the linear regime to hold. In dimensional terms, this corresponds to

47 $$\begin{aligned} {\tilde{t}}\gg \dfrac{{\tilde{K}}_4^{(2)}}{{\tilde{k}}^{(2)}}. \end{aligned}$$

#### Depletion regime

As *t* increases further, the substrate depletion and the reversible reaction from NADH to $\hbox {NAD}^{+}$ start to affect the problem at leading order. To investigate this, we make the scalings $t = T/\epsilon$ and $S_6= s_6/\epsilon$. Then, the leading-order version of (15) is

48a $$\begin{aligned} \dfrac{\mathrm {d} s_1}{\mathrm {d} T}&= - a\left( \left( \dfrac{s_1}{K_1^{(1)}+ s_1}\right) \left( \dfrac{s_2}{K_2^{(1)}+ s_2}\right) - s_4+ \rho \left( \dfrac{s_1}{K_{1}^{(-2)}+ s_1}\right) S_6\right) , \end{aligned}$$

48b $$\begin{aligned} \dfrac{\mathrm {d} s_2}{\mathrm {d} T}&= - b\left( \dfrac{s_1}{K_1^{(1)}+ s_1}\right) \left( \dfrac{s_2}{K_2^{(1)}+ s_2}\right) , \end{aligned}$$

48c $$\begin{aligned} 0&= \left( \dfrac{s_1}{K_1^{(1)}+ s_1}\right) \left( \dfrac{s_2}{K_2^{(1)}+ s_2}\right) - s_4+ \rho \left( \dfrac{s_1}{K_{1}^{(-2)}+ s_1}\right) S_6, \end{aligned}$$

48d $$\begin{aligned} \dfrac{\mathrm {d} s_5}{\mathrm {d} T}&= - c\left( s_4- \rho \left( \dfrac{s_1}{K_{1}^{(-2)}+ s_1}\right) S_6\right) , \end{aligned}$$

48e $$\begin{aligned} \dfrac{\mathrm {d} S_6}{\mathrm {d} T}&= s_4- \rho \left( \dfrac{s_1}{K_{1}^{(-2)}+ s_1}\right) S_6. \end{aligned}$$

**Fig. 9:**
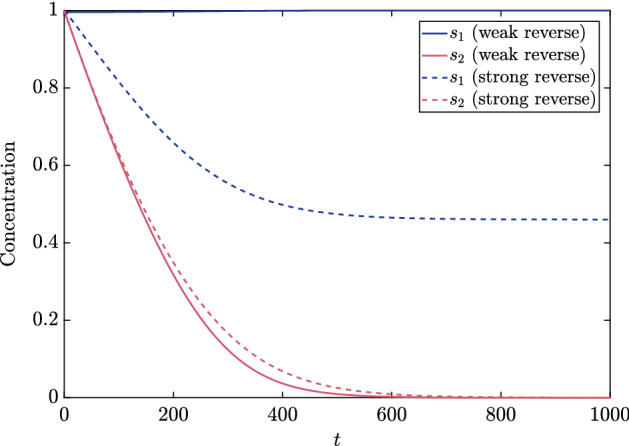
A comparison of the substrate depletion between the strong and weak reverse reactions, obtained from numerical solutions to the full system (15). We use the parameter values in Table 3, with ${\tilde{\alpha }}= {\tilde{\beta }}= 2.5$ mM, $k^{(-2)}= \epsilon K= 5 \times 10^{-4}$ for the weak reverse reaction and $k^{(-2)}= 1/K= 20$ for the strong reverse reaction

While (48) may appear complicated at first glance, it reduces readily to allow an implicit analytic solution for the system. To see this, we first use (48c) to reduce (48a) and deduce that

49a $$\begin{aligned} s_1= 1 \end{aligned}$$

at leading order over this timescale. Using (49a) in (48b) yields a separable ODE for $s_2$, with implicit solution

49b $$\begin{aligned} s_2- 1 + K_2^{(1)}\log s_2= - \dfrac{bT}{K_1^{(1)}+ 1}, \end{aligned}$$

which can also be written in terms of the Lambert *W* function. This tells us that the leading-order substrate concentration will start to decrease before the pyruvate (Fig. 9), even if the substrate is saturating *i.e.* $K_2^{(1)}\rightarrow 0$. Finally, forming linear combinations of (48b)–(48e) to yield conserved quantities allows us to deduce that

49c $$\begin{aligned} s_4&= \left( \dfrac{1}{K_1^{(1)}+ 1}\right) \left( \dfrac{s_2}{K_2^{(1)}+ s_2}\right) + \rho \left( \dfrac{1}{K_{1}^{(-2)}+ 1}\right) \left( \dfrac{1 - s_2}{b}\right) , \end{aligned}$$

49d $$\begin{aligned} s_5&= 1 - \dfrac{c}{b} \left( 1 - s_2\right) , \end{aligned}$$

49e $$\begin{aligned} S_6&= \dfrac{1 - s_2}{b}. \end{aligned}$$

From our analytic solutions, we are able to infer that the weak reverse reaction only affects the amount of $s_4$ in the system over this timescale; the other chemicals are independent of the reverse reaction despite it appearing at leading order. This change of scaling affects the asymptotic analysis of the depletion dynamics between the strong and weak reverse reaction cases; care is needed in scaling appropriately if taking $k^{(-2)}\rightarrow 0$ in (37)–(41).

We are able to deduce the long-time dynamics in terms of the implicit solution for $s_2$ given in (49b). As the experimental calculation for the reaction velocity is taken when the growth of NADH is linear, it is helpful to note when and how this breaks down in this long-time limit. To do this, we expand (49b) for small time to note that

50 $$\begin{aligned} s_6\sim V T \left( 1 - \dfrac{bK_2^{(1)}V}{2 \left( 1 + K_2^{(1)}\right) }T\right) \quad \text {as } T \rightarrow 0^{+}. \end{aligned}$$

Moving back to dimensional quantities, we find that

51 $$\begin{aligned} {\tilde{s}}_6\sim {\tilde{k}}^{(1)}\dfrac{{\tilde{\alpha }}}{{\tilde{K}}_1^{(1)}+ {\tilde{\alpha }}} \dfrac{{\tilde{\beta }}}{{\tilde{K}}_2^{(1)}+ {\tilde{\beta }}} {\tilde{t}}\left( 1 - \dfrac{{\tilde{k}}^{(1)}}{2} \dfrac{{\tilde{\alpha }}}{{\tilde{K}}_1^{(1)}+ {\tilde{\alpha }}} \dfrac{{\tilde{K}}_2^{(1)}}{\left( {\tilde{K}}_2^{(1)}+ {\tilde{\beta }}\right) ^2} {\tilde{t}}\right) , \end{aligned}$$

for

52 $$\begin{aligned} \dfrac{K_4^{(2)}}{{\tilde{k}}^{(2)}} \ll {\tilde{t}}\ll \dfrac{({\tilde{K}}_1^{(1)}+ {\tilde{\alpha }}) ({\tilde{K}}_2^{(1)}+ {\tilde{\beta }})^2}{\alpha {\tilde{k}}^{(1)}{\tilde{K}}_2^{(1)}}. \end{aligned}$$

The simplest way to make this constraint less limiting is to have as small a value of $\epsilon = {\tilde{k}}^{(1)}/{\tilde{k}}^{(2)}$ as possible. Another way is to increase ${\tilde{\beta }}$, though we note that this also has the effect of making it difficult to determine ${\tilde{K}}_2^{(1)}$. Therefore, to minimize the correction term (and hence increase the duration of time at which the NADH growth is linear, thus reducing experimental error), we should decrease ${\tilde{\alpha }}$ and increase ${\tilde{\beta }}$. The former of these is perhaps a little surprising, though we note that we must also ensure that pyruvate depletion does not affect anything at leading order, so ${\tilde{\alpha }}\gg \epsilon {\tilde{K}}_4^{(2)}\approx 0.05$ mM must also hold.

There will be further asymptotic regions and depletion dynamics at play here at longer timescales, such as if $s_5$ becomes of $\textit{O}(\delta )$. However, these additional regions are of limited relevance to our analysis here.

## Guide to using our theoretical results

We now summarize the main results from Sects. 3.1 and 3.2, and give a step-by-step guide of how to use these to characterize enzymes. We have found that a linear-growth regime for NADH is possible for a strong reverse reaction, but this regime can be quite short and is sandwiched between two nonlinear-growth regimes. The NADH concentration in the linear regime is given in (31) in terms of dimensional quantities. Although the linear-growth regime in the weak reverse reaction case is also sandwiched between two nonlinear-growth regimes, it lasts for a much longer duration in this case and so results from the linear regime are easier to use in practice.

**Fig. 10:**
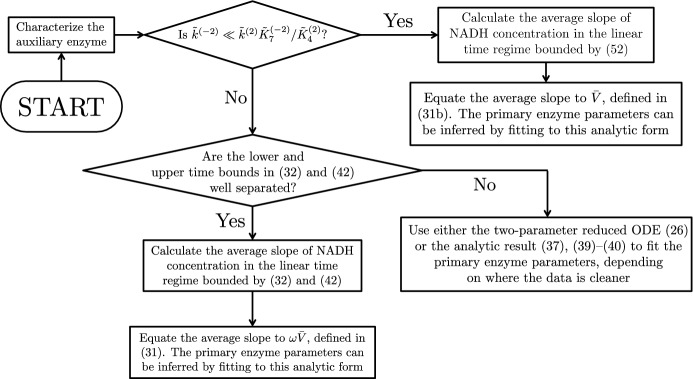
A flow chart summarising how to use the main results of this paper. Further details of each step are provided in the main text of Sect. 4

Due to our analytic asymptotic results, we are able to deduce upper and lower time constraints for the linear regimes, which we give in (33) and (43) in terms of dimensional quantities for the strong reverse reaction case and in (52) for the weak reverse reaction case. We are also able to obtain an implicit closed-form solution for the NADH concentration away from this linear regime, which would allow the unknown quantities to be inferred through a nonlinear parameter fitting, if the linear regime was too short for accurate measurements to be obtained. We now provide a step-by-step guide to using our results with time data for the concentration of the indicator chemical in each case, using different values of the initial substrate concentrations ${\tilde{\alpha }}$ and ${\tilde{\beta }}$. We also present a summary of these guidelines as a flow chart in Fig. 10, and note that a summary of the solutions we derive in this paper in provided in Table 4.

*Step 1* Characterize the auxiliary enzyme.

*Step 2* Determine ${\tilde{k}}^{(-2)}$, the maximum velocity of the reverse reaction for the auxiliary enzyme. If ${\tilde{k}}^{(-2)}$ is significantly smaller than ${\tilde{k}}^{(2)}{\tilde{K}}_{7}^{(-2)}/{\tilde{K}}_4^{(2)}$ (say, two-to-three orders of magnitude), proceed to Sect. 4.1. If not, proceed to Sect. 4.2.

### Using the weak reverse reaction results

In this case, the NADH concentration should exhibit linear growth for a significant period of time, with the relevant bounds given in (52). We are able to reduce the lower bound in (52) by adding more of the auxiliary enzyme, thereby reducing the lag period. Additionally, although the upper bound in (52) contains the parameters we are trying to infer, we can see that adding less of the primary enzyme will increase it, extending the length of time until the depletion dynamics kick in.

From the experimental data in the linear growth regime, the procedure is to calculate the average slope of NADH concentration. This slope is equal to ${\tilde{V}}$, defined in (32). The kinetic parameters of the primary enzyme can be inferred by fitting the average slope obtained from the experimental data to the expression we give for ${\tilde{V}}$ in (32), using nonlinear data-fitting to minimize the sum of the difference between the data and functional form.

### Using the strong reverse reaction results

In this case, the NADH concentration should exhibit linear growth for some period of time, with the relevant bounds given in (33) and (43). While the upper bound of (43) can be extended by adding less of the primary enzyme, it is not necessarily true in this case that adding more of the auxiliary enzyme will decrease the lower bound of (33). If the linear regime is able to be observed for an appropriate length of time, then the appropriate approach is to calculate the average slope of NADH concentration from the experimental data in the linear growth regime. This slope is equal to ${\tilde{V}}\omega$, defined in (31). Here, $\omega$ is a function of the kinetic parameters of the auxiliary enzyme and the initial chemical concentrations, whereas ${\tilde{V}}$ depends on the kinetic parameters of the primary enzyme and the initial chemical concentrations. Hence, the kinetic parameters of the primary enzyme can be inferred by fitting the average slope obtained from the experimental data to the expression we give for ${\tilde{V}}\omega$ in (32), using nonlinear data-fitting to minimize the distance between the data and functional form.

If the linear regime is *not* able to be observed for an appropriate length of time, there are two options, depending on whether better data is available for ${\tilde{t}}= \textit{O}({\tilde{K}}_4^{(2)}/{\tilde{k}}^{(2)})$ or for ${\tilde{t}}= \textit{O}({\tilde{\alpha }}/{\tilde{k}}^{(1)})$. In the former case, one should use the results from Sect. 3.1, where the data-fitting has been reduced to the single ODE (21). In terms of dimensional quantities, the ODE (21) can be re-written as

53 $$\begin{aligned} \dfrac{\mathrm {d} {\tilde{s}}_6}{\mathrm {d} {\tilde{t}}} = \left( \dfrac{{\tilde{V}}{\tilde{k}}^{(2)}}{{\tilde{K}}_4^{(2)}}\right) {\tilde{t}}- \left( \dfrac{{\tilde{k}}^{(2)}}{{\tilde{K}}_4^{(2)}}\right) {\tilde{s}}_6- \left( \dfrac{{\tilde{k}}^{(-2)}}{{\tilde{K}}_{7}^{(-2)}} \dfrac{{\tilde{\alpha }}}{{\tilde{K}}_{1}^{(-2)}+ {\tilde{\alpha }}}\right) \dfrac{ {\tilde{s}}_6^2}{{\tilde{K}}_{6}^{(-2)}+ {\tilde{s}}_6}, \quad {\tilde{s}}_6(0) = 0. \end{aligned}$$

While there are four different parameter groupings in (53), the kinetic parameters of the primary enzyme are all contained within ${\tilde{V}}$, as described in (32). Therefore, the data fitting to this ODE is likely to be significantly simpler than data-fitting to the initial system of ODEs (1). If better data is instead available for ${\tilde{t}}= \textit{O}({\tilde{\alpha }}/{\tilde{k}}^{(1)})$, then the results of Sect. 3.1.3 should be used. Here, the functional form for the NADH concentration is given by (38), (40), and (41). In this case, the data-fitting should involve minimizing the sum of the difference between the time data and the functional form.

### Comparison with a naive nonlinear solver

**Fig. 11:**
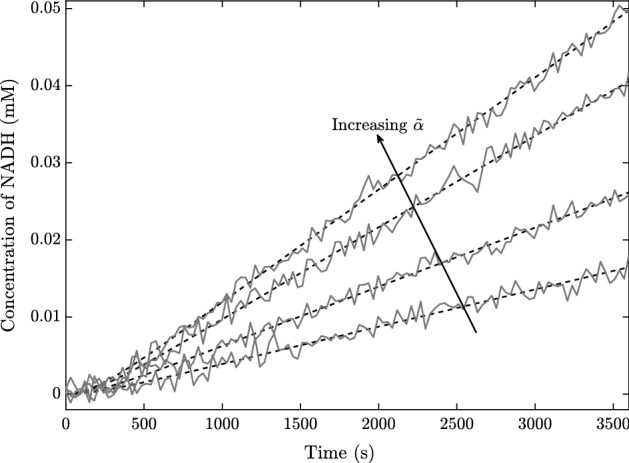
An example of the ‘true’ time courses generated from the system (10)–(11), with added Gaussian noise of mean 0 $\upmu$M and standard deviation $1 \, \upmu$M. The data without noise is indicated by the dashed lines. We use the parameter values from Table 2, as well as ${\tilde{k}}^{(1)}= 0.02 \, \upmu$M $\hbox {s}^{-1}$, ${\tilde{k}}^{(2)}= 10 \, \upmu$M $\hbox {s}^{-1}$, ${\tilde{k}}^{(-2)}= 10 \, \upmu$M $\hbox {s}^{-1}$, ${\tilde{K}}_1^{(1)}= 1.5$ mM, ${\tilde{K}}_2^{(1)}= 1$ mM, ${\tilde{\beta }}= 50$ mM, and ${\tilde{\gamma }}= 50$ mM. The four different curves correspond to ${\tilde{\alpha }}= 0.5$, 1, 2.5, 5 mM

We now compare the asymptotic method we describe in this paper with a naive nonlinear parameter fitting. To do this, we first generate ‘true’ time courses for NADH from the system (10)–(11) for four different values of $${\tilde{\alpha }}$$, then add Gaussian noise with mean 0 $$\upmu $$M and standard deviation $$\sigma $$$$\upmu $$M, and we use $$\sigma $$ as a control parameter. An example of these time courses with $$\sigma = 1$$ is given in Fig. 11. The asymptotic parameter fitting is carried out by fitting the asymptotic solution (31) to the true time courses using the MATLAB function ‘lsqcurvefit’ for the parameters $${\tilde{k}}^{(1)}$$ and $${\tilde{K}}_1^{(1)}$$. The naive nonlinear parameter fitting is carried out using the same MATLAB function ‘lsqcurvefit’ on the ODE system (10)–(11), fitting the parameter values $${\tilde{k}}^{(1)}$$, $${\tilde{K}}_1^{(1)}$$, and $${\tilde{K}}_2^{(1)}$$. However, as we generate the true time courses using a large value of $${\tilde{\beta }}$$ in order to focus on $${\tilde{K}}_1^{(1)}$$ rather than $${\tilde{K}}_2^{(1)}$$, we ignore the value of $${\tilde{K}}_2^{(1)}$$ generated from this.

**Fig. 12:**
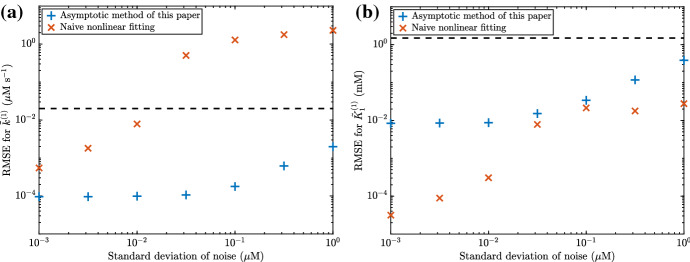
A comparison between the asymptotic method presented in this paper, and a naive nonlinear fit of the system (10)–(11). We show the root mean squared error (RMSE) for both methods in estimating the parameters **a** $${\tilde{k}}^{(1)}$$ and **b** $${\tilde{K}}_1^{(1)}$$. The dashed line shows the value of the parameter being estimated, so a RMSE well below this line signifies good accuracy whereas a RMSE near or above the line signifies poor accuracy. We generate the ‘true’ data from the system (10)–(11), then add Gaussian noise with mean $$0 \, \upmu $$M and standard deviation indicated on the *x*-axis. Each marker represents the RMSE calculated from 100 total realisations. We use the parameter values from Table 2, as well as $${\tilde{k}}^{(1)}= 0.02 \, \upmu $$M $$\hbox {s}^{-1}$$, $${\tilde{k}}^{(2)}= 10 \, \upmu $$M $$\hbox {s}^{-1}$$, $${\tilde{k}}^{(-2)}= 10 \, \upmu $$M $$\hbox {s}^{-1}$$$${\tilde{K}}_1^{(1)}= 1.5$$ mM, $${\tilde{K}}_2^{(1)}= 1$$ mM, $${\tilde{\beta }}= 50$$ mM, and $${\tilde{\gamma }}= 50$$ mM. We also use $${\tilde{\alpha }}= 0.5$$, 1, 2.5, 5 mM to generate four different ‘true’ time course curves for each different standard deviation of noise

In general, the naive nonlinear fit is worse than the asymptotic fit in determining ${\tilde{k}}^{(1)}$ and better than the asymptotic fit in determining ${\tilde{K}}_1^{(1)}$ (Fig. 12). However, the asymptotic fit appears to be more consistent in its predictions than the naive nonlinear fit, as the naive nonlinear fit can predict wildly incorrect values of ${\tilde{k}}^{(1)}$ even for small noise. Moreover, we note that the computational time required for the naive nonlinear fit is around 1000 times larger than for the asymptotic method.

## Application to experimental data

The above concludes our mathematical analysis of the problem. To showcase how these results can be used in practice, we performed enzyme assays to characterize an enzyme for two different substrates using a previously uncharacterized putative omega-aminotransferase, cloned and purified in a heterologous *Escherichia coli* host. The aminotransferase is CnAptA (UniProtKB Q0KEZ8), named here for the first time in the literature and identified in the genome of *Cupriavidus necator* H16, an industrially relevant, facultative chemolithoautotrophic chassis microorganism of the Synthetic Biology Research Centre (SBRC-Nottingham). We describe the experimental protocol used to purify this enzyme in “Appendix B”.

### Enzyme assay protocol

For these assays, the corresponding reaction network is shown in Fig. 1. The primary enzyme is CnAptA, which converts pyruvate and an additional omega-amino acid substrate into a corresponding aldehyde and alpha-L-alanine. Our assay solution also included beta-nicotinamide adenine dinucleotide sodium salt ($\hbox {NAD}^{+}$) as an electron acceptor for the auxiliary enzyme reaction, and we monitored the formation of NADH over time, produced from the auxiliary reaction. We used a commercially available alanine dehydrogenase (A-DH) from *Bacillus cereus* (alanine dehydrogenase, recombinant; CAS-No 9029-06-5, Sigma-Aldrich Company Ltd.) as the auxiliary enzyme, and we performed the experiment at pH 10, the pH condition recommended by the supplier of this auxiliary enzyme, this condition resulting in an undetectable reverse reaction. Our analysis suggests that the primary enzyme will be easier to characterize, since the bounds on the validity of the linear regime are less severe. Hence, we can either use the sub-limit as $\omega \rightarrow 1$ of the results in the distinguished limit (31), or we can use the sub-limit directly deduced in Sect. 3.2. The approach for this is summarised in Sects. 4 and 4.1. Performing these assays at different acidities, which may be more relevant for certain bacterial cell environments, would result in a strong reverse reaction, which could be dealt with using the analytic representation for the distinguished limit given in (31). The approach for these cases is summarised in Sects. 4 and 4.2.

We performed the enzyme assays in 96 well microtiter flat bottom transparent plates (Costar^®^ Sigma-Aldrich). The reaction mixture contained different concentrations of the substrate, 2.5 mM $\hbox {NAD}^{+}$, 5 mM pyruvate, 5 $\upmu$l A-DH [0.28 mg/ml], 5 $\upmu$M of pyridoxal 5$'$-phosphate (PLP), and 5 $\upmu$l of CnAptA [0.0245 mg/ml]. We then used an appropriate amount of sodium carbonate buffer to obtain a final volume of 200 $\upmu$l. The two different substrates we used were 3-aminobutanoic acid (Sigma-Aldrich 97%, CAS No 541-46-6) and 5-aminovalerate (ACROS Organics, 97%. CAS No 660-88-8).

**Fig. 13:**
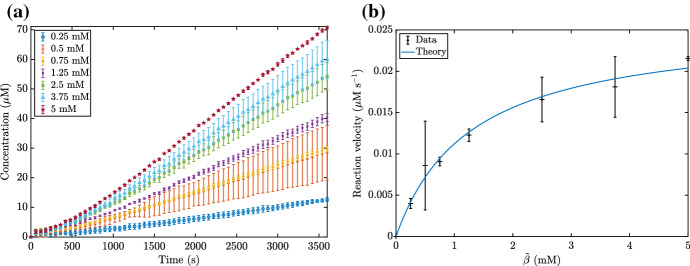
**a** The time course data for NADH production with the enzyme CnAptA and substrate 3-aminobutanoic acid, using different concentrations of the latter. **b** The average reaction velocity at different substrate concentrations, and the predicted reaction velocity using our nonlinear data fitting to obtain values of ${\tilde{k}}^{(1)}= 0.026 \, \upmu$M $\hbox {s}^{-1}$ and ${\tilde{K}}_2^{(1)}= 1.3$ mM, each to two significant figures. The error bars on the figures correspond to one standard deviation of the data

**Fig. 14:**
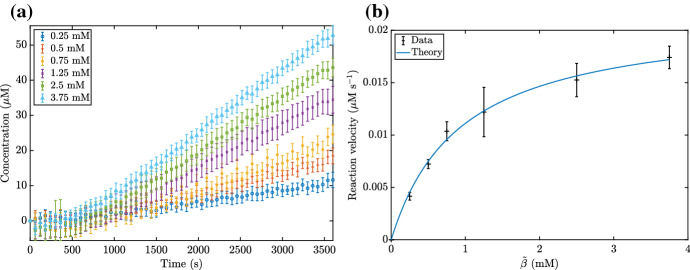
**a** The time course data for NADH production with the enzyme CnAptA and substrate 5-aminovalerate, using different concentrations of the latter. **b** The average reaction velocity at different substrate concentrations, and the predicted reaction velocity using our nonlinear data fitting to obtain values of ${\tilde{k}}^{(1)}= 0.021 \, \upmu$M $\hbox {s}^{-1}$ and ${\tilde{K}}_2^{(1)}= 0.93$ mM. The error bars on the figures correspond to one standard deviation of the data

To detect the NADH present in the solution, we measured the light absorbance at 340 nm using a TECAN $\hbox {Infinite}^{\textregistered }$ M1000 PRO plate reader, at $30\ ^{\circ }\hbox {C}$, the absorbance being linearly proportional to the concentration of NADH in the solution. We monitored the light absorbance at 340 nm at 60 s intervals for 1 h. After subtracting off the base level of absorbance at the start of each experiment and the absorbance increase monitored in the control experiment with no substrate, we converted these clean absorbance data into an NADH concentration, shown for each substrate in Figs. 13a and 14a. We expect our TECAN data to be accurate to within a few $\upmu$M, and this error margin is why there sometimes appears to be ‘negative’ concentrations for early time. As our analysis predicts, there is a clear linear regime after a lag period. We obtained a constant reaction velocity from this linear regime, using the average slope between 1800 and 3600 s. From this constant reaction velocity, we used a nonlinear data fit to the functional form of the solution (31)–(32) with $\omega \rightarrow 1$ (as discussed in Sects. 4 and 4.1) by minimizing the sum of the difference between the data and functional form squared, using the in-built function fminsearch in MATLAB, a simplex search method for finding minima (Lagarias et al. 1998). From Figs. 13b and 14b, we see that our nonlinear fits generally provide accurate representations of the data. For the enzyme CnAptA, our nonlinear fit predicts values of ${\tilde{k}}^{(1)}= 0.026 \, \upmu$M $\hbox {s}^{-1}$ and ${\tilde{K}}_2^{(1)}= 1.3$ mM for the substrate 3-aminobutanoic acid and values of ${\tilde{k}}^{(1)}= 0.021 \, \upmu$M $\hbox {s}^{-1}$ and ${\tilde{K}}_2^{(1)}= 0.93$ mM for the substrate 5-aminovalerate, assuming that the levels of pyruvate we use are saturating. In terms of the catalytic efficiencies, we see that ${\tilde{k}}^{(1)}/{\tilde{K}}_2^{(1)}= 2.0 \times 10^{-5} \, \text {s}^{-1}$ for 3-aminobutanoic acid and ${\tilde{k}}^{(1)}/{\tilde{K}}_2^{(1)}= 2.3 \times 10^{-5} \, \text {s}^{-1}$ for 5-aminovalerate. Therefore, we note that while 5-aminovalerate has a higher affinity (a lower ${\tilde{K}}_2^{(1)}$) to and catalytic efficiency for CnAptA than 3-aminobutanoic acid, the maximum reaction velocity is higher for 3-aminobutanoic acid than for 5-aminovalerate. However, these differences are fairly small - there is not a large difference in enzyme behaviour between the two substrates.

## Discussion

We have used singular perturbation theory to determine how to infer the kinetic parameters of the primary enzyme from the measured NADH concentration in a coupled enzyme assay, with reaction network shown in Fig. 1. In particular, this will allow putative aminotransferases to be quickly characterized using coupled enzyme assays. Our analysis also allows us to obtain estimates for when we expect to see a linear growth in NADH concentration, the typical regime measured in enzyme assays since it allows a quick sanity check of experimental results and is much easier to fit to data. We have outlined how to use our theoretical results to infer the kinetic parameters of the primary enzyme from experimental data in Sect. 4, where we also compared our method with a a naive nonlinear fit. Moreover, we have used our results to characterize a novel putative aminotransferase for two different substrates in Sect. 5.

Our analysis shows that there are two distinguished asymptotic limits in the system: the important one for NADH (the measurable chemical) being the strong reverse reaction case, considered in Sect. 3.1; the other is the weak reverse reaction case, considered in Sect. 3.2. We demonstrate that all the information that can be inferred from the NADH concentration in the weak reverse reaction case is a sub-limit of the strong reaction case, even though there are differences in the concentrations of alpha-L-alanine, an intermediate chemical, between these two cases. In our analysis, we exploit the smallness of three dimensionless parameters: $\epsilon$, $\delta$, and $K$, with a focus on analysing how the asymptotic size of $k^{(-2)}$ affects the system behaviour and treating all other parameters in the system as $\textit{O}(1)$ to retain their generality. As the parameters $\epsilon$ and $\delta$ can always be made to be small through experimental choice, and we also present results for $K= \textit{O}(1)$ in “Appendix C” (which shows a slight change in the depletion regimes), our work represents a comprehensive asymptotic analysis of the possible behaviours of the non-standard coupled enzyme assay we consider in this paper.

Additionally, we note that our results suggest that one cannot deduce whether the strong reaction case pertains simply by looking for a change in reaction velocity when the amount of auxiliary enzyme is varied. This can be seen by (31), where we see that the only two variables that involve the experimentally controlled auxiliary enzyme concentration, ${\tilde{k}}^{(2)}$ and ${\tilde{k}}^{(-2)}$, appear as a ratio, thus removing any dependence on this enzyme concentration. Thus, it is not always immediately clear from observations whether one is in the weak or strong reverse reaction regime, unless the auxiliary reaction has previously been completely characterized. Due to the weak reverse reaction being a sub-limit of the strong for the experimentally measurable NADH concentration, as discussed above, we advise working with the results derived in the strong reverse reaction regime in Sect. 3.1.

In the derivation of our model, we assume that the kinetic parameters of the putative enzyme are unchanging over time. This may not be valid for enzymes that are unstable or suffer a loss of activity over time, for example through incubation in a nonoptimal buffer at certain temperatures. Accounting for this change in enzyme activity would require additional information about the type and effect of the degradation occurring.

Although it would be possible to perform a brute force fitting of the kinetic parameters of the primary enzyme using the governing equations (10), such an approach would be time-consuming and would not provide the general analytic expressions we derive through our asymptotic analysis. Moreover, we are able to use our analytic expressions to estimate when we will be in the linear growth regime, and for how long we expect this regime to last, in terms of the experimental parameters. This provides a useful sanity check when it comes to interpreting the data.

Historically, the measured reaction velocity is often converted into kinetic parameters for the enzyme using a Lineweaver–Burke plot (Lineweaver and Burk 1934). However, this procedure is generally not the best way to calculate these values as it amplifies noise in the measurements (Fell 1997). Nonlinear data fitting is orders of magnitude better than it was at the time of Lineweaver and Burk, so it is much more accurate nowadays to fit the data to analytic forms such as those we have deduced using singular perturbation theory. For the case we consider in this paper, measurements with varying initial substrate concentrations give us a two-dimensional array of data from which to fit three variables. Additionally, choosing one of these substrate concentrations to be much larger than the estimated Michaelis constant ${\tilde{K}}_1^{(1)}$ or ${\tilde{K}}_2^{(1)}$ reduces the problem to a one-dimensional array of data from which to fit two variables.

While we have considered a specific type of non-standard coupled enzyme assay here, the asymptotic techniques we use are far from restricted to this system. We hope that the techniques used in this paper will be applied to different reaction networks, to help characterize enzymes that can only be assayed in non-standard reaction network topologies.

## Figures and Tables

**Fig. 15:**
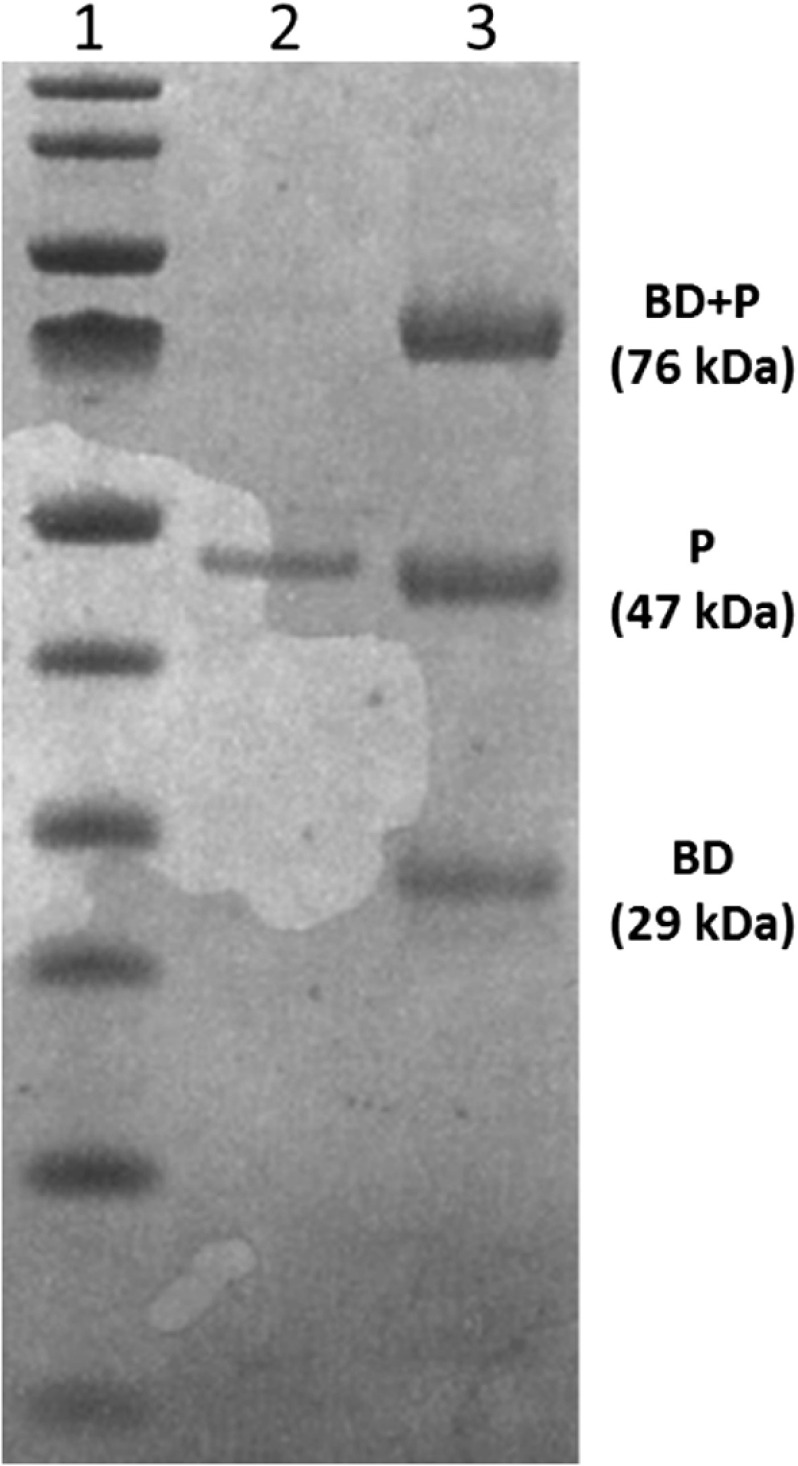
The ColorPlus^TM^ pre-stained protein ladder (10–230 kDa, Lane 1), the concentrated CnAptA protein after on column cleavage (Lane 2, 47 kDa) and 10 $\upmu$l of the resin removed after washing and cleavage of the target protein (Lane 3) was loaded onto a NuPAGE® Novex 4–12% Bis-Tris SDS-PAGE Gel and run for 2 h at 150 V in NuPAGE® MES SDS Running Buffer. Protein bands were visualised using Coomassie Blue staining. BD+P: intein tagged CnAptA, P:CnAPTA after intein tag cleavage, BD: cleaved intein tag

## Distinguished asymptotic limits of equation (26)

In this Appendix, we investigate the distinguished asymptotic limits of (26), thus allowing us to characterize the possible behaviours of its solution.

### Case I

The first distinguished limit we discuss (Region I in Fig. 7) occurs when ${\bar{V}} \ll 1$ and $\gamma = \textit{O}(1)$ for the ODE (26). Here, there are two different timescales in the problem: $t = \textit{O}(1)$ and $t = \textit{O}(1/{\bar{V}})$, for which $Y = \textit{O}({\bar{V}})$ and $Y = \textit{O}(1)$, respectively; the limit is distinguished only over the latter. Therefore, our brief analysis of the earlier timescale consists simply of the asymptotic solution

54 $$\begin{aligned} Y \sim {\bar{V}} \left( t - 1 +e^{-t}\right) . \end{aligned}$$

For the later timescale, the leading-order governing equation is

55 $$\begin{aligned} 0 = {\bar{V}} t - Y - \gamma \dfrac{Y^2}{1 + Y} \end{aligned}$$

and hence we are in the full quasi-steady case. The equation (55) has solution

56 $$\begin{aligned} Y = \dfrac{{\bar{V}} t - 1 + \sqrt{({\bar{V}} t + 1)^2 + 4 {\bar{V}} \gamma t}}{2(\gamma + 1)}, \end{aligned}$$

which matches appropriately to the earlier-time solution. We note that the large-time limit of (56) is $Y \sim {\bar{V}} t/(\gamma + 1)$. This is different to the large-time limit of (54) (when $t = \textit{O}(1)$), which is $Y \sim {\bar{V}} t$. Therefore, there are two different linear regimes here, and one should take great care in this distinguished limit to ensure that one is aware of in which linear regime the measurements are being taken: failure to do so could result in a significant error of interpretation.

When $\gamma \rightarrow \infty$ in this distinguished limit, we move into sub-limit A (Fig. 7). Here, the earliest timescale is the same as (54), but the later timescale splits into two, the earlier with $t = \textit{O}(1/({\bar{V}} \gamma ))$ and $Y = \textit{O}(1/\gamma )$, the later with $t = \textit{O}(\gamma /{\bar{V}})$ and $Y = \textit{O}(1)$, as could be inferred from the large-$\gamma$ limit of (56). On the timescale $t = \textit{O}(1/({\bar{V}} \gamma ))$, the solution is

57 $$\begin{aligned} Y = \dfrac{ \sqrt{1 + 4 {\bar{V}} \gamma t} - 1}{2\gamma }, \end{aligned}$$

and on the timescale $t = \textit{O}(\gamma /{\bar{V}})$

58 $$\begin{aligned} Y = \dfrac{{\bar{V}} t + \sqrt{({\bar{V}} t)^2 + 4 {\bar{V}} \gamma t}}{2 \gamma }. \end{aligned}$$

When $\gamma \rightarrow 0$ in this distinguished limit, we move into sub-limit D (Fig. 7). Here, the full leading-order solution in the later timescale is simply the large-time limit of the earlier timescale. Hence, the leading-order solution is simply (54), uniformly valid for all time.

### Case II

The next distinguished limit (Region II in Fig. 7) occurs when ${\bar{V}} \ll 1$ and $\gamma \gg 1$, with ${\bar{V}} \gamma = \textit{O}(1)$, in the ODE (26). Here, there are two different timescales in the problem: $t = \textit{O}(1)$ and $t = \textit{O}(\gamma ^2)$, for which $Y = \textit{O}(1/\gamma )$ and $Y = \textit{O}(1)$, respectively; the limit is distinguished only over the earlier of these. Taking $Y = {\bar{Y}} / \gamma$, the leading-order equation for the earlier timescale is

59 $$\begin{aligned} \frac{\mathrm {d} {\bar{Y}}}{\mathrm {d} t} = {\bar{V}} \gamma t - {\bar{Y}} - {\bar{Y}}^2, \quad {\bar{Y}}(0) = 0. \end{aligned}$$

The Riccati equation (59) has solution

60a $$\begin{aligned} {\bar{Y}}&= \left( {\bar{V}} \gamma \right) ^{1/3} \dfrac{{{\,\mathrm{Bi}\,}}'[T(t)] + \alpha {{\,\mathrm{Ai}\,}}'[T(t)]}{{{\,\mathrm{Bi}\,}}[T(t)] + \alpha {{\,\mathrm{Ai}\,}}[T(t)]} - \dfrac{1}{2}, \end{aligned}$$

60b $$\begin{aligned} \alpha&= \dfrac{2 \left( {\bar{V}} \gamma \right) ^{1/3} {{\,\mathrm{Bi}\,}}'[T(0)] - {{\,\mathrm{Bi}\,}}[T(0)]}{ {{\,\mathrm{Ai}\,}}[T(0)] - 2 \left( {\bar{V}} \gamma \right) ^{1/3} {{\,\mathrm{Ai}\,}}'[T(0)]}, \end{aligned}$$

60c $$\begin{aligned} T(t)&= \dfrac{1}{4 \left( {\bar{V}} \gamma \right) ^{2/3}} + \left( {\bar{V}} \gamma \right) ^{1/3} t, \end{aligned}$$

where ${{\,\mathrm{Ai}\,}}(z)$ and ${{\,\mathrm{Bi}\,}}(z)$ are the standard Airy functions. The solution (60a) exhibits the following large-*t* behaviour

61 $$\begin{aligned} {\bar{Y}} \sim \sqrt{{\bar{V}} \gamma t} \quad \text {as } t \rightarrow \infty . \end{aligned}$$

The later timescale in this problem occurs when $t = \textit{O}(\gamma ^2) = \textit{O}(1/{\bar{V}}^2)$ and $Y = \textit{O}(1)$. From (26), this timescale yields the following leading-order equation

62 $$\begin{aligned} {\bar{V}} t = \gamma \dfrac{Y^2}{1 + Y}, \end{aligned}$$

with solution

63 $$\begin{aligned} Y = \dfrac{{\bar{V}} t + \sqrt{({\bar{V}} t)^2 + 4 {\bar{V}} \gamma t}}{2 \gamma }. \end{aligned}$$

This exhibits the large-*t* behaviour

64 $$\begin{aligned} Y \sim {\bar{V}} t/\gamma \quad \text {as } {\bar{t}} \rightarrow \infty . \end{aligned}$$

Hence, we may deduce that in this regime the variable *Y* initially behaves as $Y \sim {\bar{V}} t^2 / 2$, then as $Y \sim \sqrt{{\bar{V}} t / \gamma }$, then finally as $Y \sim {\bar{V}} t / \gamma$, and therefore exhibits at least two points of inflection, as well as three distinct power laws.

The solutions of the distinguished limit in Case II tend to those of the sub-limit A (Fig. 7) as ${\bar{V}} \gamma \rightarrow 0$. The early timescale, when $t = \textit{O}(1)$ with solution (60a), splits into two, with solutions (54) and (57). The late timescale solution (63) is the same as in sub-limit A, as given in (58).

As ${\bar{V}} \gamma \rightarrow \infty$, we move into sub-limit B (Fig. 7). There are two different timescales in this sub-limit: $t = \textit{O}(1/({\bar{V}} \gamma )^{1/3})$ and $t = \textit{O}(\gamma /{\bar{V}})$, for which $Y = \textit{O}(({\bar{V}}/\gamma ^2)^{1/3})$ and $Y = \textit{O}(1)$, respectively. For the first, $t = \textit{O}(1/({\bar{V}} \gamma )^{1/3})$, the solution (60a) reduces to

65 $$\begin{aligned} Y = \left( \dfrac{{\bar{V}}}{\gamma ^2}\right) ^{1/3} \dfrac{{{\,\mathrm{Bi}\,}}'\left[ \left( \gamma {\bar{V}}\right) ^{1/3} t\right] + \sqrt{3} {{\,\mathrm{Ai}\,}}'\left[ \left( \gamma {\bar{V}}\right) ^{1/3} t\right] }{{{\,\mathrm{Bi}\,}}\left[ \left( \gamma {\bar{V}}\right) ^{1/3} t\right] + \sqrt{3} {{\,\mathrm{Ai}\,}}\left[ \left( \gamma {\bar{V}}\right) ^{1/3} t\right] }, \end{aligned}$$

For the second, $t = \textit{O}(\gamma /{\bar{V}})$, the leading-order solution is the same as for Case II, given by (63).

### Case III

The next distinguished limit (Region III in Fig. 7) occurs when ${\bar{V}} \gg 1$ and $\gamma \gg 1$, with ${\bar{V}} /\gamma ^2 = \textit{O}(1)$, in the ODE (26). Here, the important timescale has $t = \textit{O}(1/\gamma ) = \textit{O}(1/{\bar{V}}^{1/2})$, for which $Y = \textit{O}(1)$. To investigate this, we make the substitution $t = \tau / \gamma$, resulting in the leading-order behaviour being described by

66 $$\begin{aligned} \frac{\mathrm {d} Y}{\mathrm {d} \tau } = \dfrac{{\bar{V}}}{\gamma ^2} \tau - \dfrac{Y^2}{1 + Y}, \quad Y(0) = 0. \end{aligned}$$

While we cannot solve (66) analytically, we note that the large-$\tau$ behaviour of (66) is $Y \sim {\bar{V}} \tau / \gamma ^2 = {\bar{V}} t / \gamma$, and that this behaviour will dominate when $t = \textit{O}(1)$. Moreover, we can show that this case reduces to the sub-limits B and C (Fig. 7) as ${\bar{V}} /\gamma ^2 \rightarrow 0$ and ${\bar{V}} /\gamma ^2 \rightarrow \infty$, respectively.

In the former sub-limit, with ${\bar{V}} /\gamma ^2 \rightarrow 0$, there are two timescales. For the earlier timescale scaling $Y \sim ({\bar{V}}/\gamma ^2)^{1/3}$ and $\tau \sim (\gamma ^2/{\bar{V}})^{1/3}$ (equivalent to $t \sim 1/(\gamma {\bar{V}})^{1/3}$) results in the reduction of (66) to a Riccati equation with solution (65). The problem for the later timescale can be obtained by scaling $\tau \sim \gamma ^2/{\bar{V}}$ (equivalent to $t \sim \gamma /{\bar{V}}$), and this has solution (63).

For the latter sub-limit, with ${\bar{V}} /\gamma ^2 \rightarrow \infty$, the important balance occurs when $\tau = \textit{O}(1)$ (equivalent to $t \sim 1/\gamma$) and $Y = \textit{O}({\bar{V}} /\gamma ^2)$, resulting in the leading-order system

67 $$\begin{aligned} \frac{\mathrm {d} Y}{\mathrm {d} \tau } = \dfrac{{\bar{V}}}{\gamma ^2} \tau - Y, \quad Y(0) = 0, \end{aligned}$$

which has solution

68 $$\begin{aligned} Y = \dfrac{{\bar{V}}}{\gamma ^2} \left( \tau - 1 + e^{-\tau } \right) = \dfrac{{\bar{V}}}{\gamma ^2} \left( \gamma t - 1 + e^{-\gamma t} \right) . \end{aligned}$$

### Case IV

The final distinguished limit (Region IV in Fig. 7) occurs when ${\bar{V}} \gg 1$ and $\gamma = \textit{O}(1)$ in the ODE (26). Here, the main balance occurs when $t = \textit{O}(1)$ and $Y = \textit{O}({\bar{V}})$. In this case, the leading-order version of the ODE (26) is

69 $$\begin{aligned} \frac{\mathrm {d} Y}{\mathrm {d} t} = {\bar{V}} t - Y - \gamma Y, \quad Y(0) = 0, \end{aligned}$$

which has solution

70 $$\begin{aligned} Y = \dfrac{{\bar{V}}}{(1 + \gamma )^2} \left[ (1 + \gamma ) t - 1 + e^{-(1 + \gamma )t} \right] . \end{aligned}$$

In the limit of $\gamma \rightarrow \infty$, the solution (70) reduces to that in sub-limit C (Fig. 7), given by (68), with the important timescale $t = \textit{O}(1/\gamma )$. In the limit of $\gamma \rightarrow 0$, the solution (70) reduces to that of sub-limit D (Fig. 7), given by (54). This completes our comprehensive asymptotic analysis of the distinguished limits of the ODE (26).

## Experimental protocol

### Bacterial strains and cultivation conditions

We purchased the bacterial strains used in this study from New England Biolabs (*E. coli* ER2566) and DSMZ-Deutsche Sammlung von Mikroorganismen und Zellkulturen GmbH DSMZ (*C. necator* H16). Lysogeny broth (LB) medium was used for cultivation of both strains. We cultivated strains carrying the pTXB1 plasmid (supplied with the $\hbox {IMPACT}^{\text {TM}}$ KIT (NEB#E6901)) in the same media supplemented with ampicillin, at a final concentration of 100 $\mu$g/ml.

### Cloning of the putative aminotransferase CnAptA

We amplified the coding region of CnAptA by PCR from the genomic DNA of *C. necator* H16 using 0.5 $\upmu$M of FwaptA (5$'$ taccatatggacgccgccaagaccgt 3$'$) and RvaptA (5$'$ tgcaggaagagcccttgatgtcctgcagcgccttct 3’) primer pairs, using the Q5^®^ High-fidelity 2X Master Mix (NEB, #M0492S) with the following PCR conditions: 3 min of denaturation at 98 $^{\circ }$C, followed by 25 cycles of 10 s denaturation at 98 $^{\circ }$C, 30 s of an annealing temperature at 72 $^{\circ }$C, and 1 min extension of at 72 $^{\circ }$C. We also added 5% DMSO to the PCR mixture due to the high GC content of the amplicon.

We digested the resulting PCR products with the *Nde*I and *Sap*I restriction enzymes and ligated into the pTXB1 expression vector supplied with the $\hbox {IMPACT}^{\text {TM}}$ KIT, which we digested with the same restriction enzymes. We introduced the resulting plasmid, pTXB1-CnAptA, into the *E. coli* ER2566 strain. The pTXB1 vector allows for the fusion of the target protein at its C-terminus to the affinity tag (chitin binding domain or CBD) via a protein splicing element. This system is suitable for the purification of the native recombinant protein in a single chromatographic step, without the use of a protease. The expression of the fusion gene is controlled by an isopropyl-beta-D-1-tiogalactopyranoside (IPTG)-inducible T7 promoter.

To overexpress the putative transaminase CnAptA, we grew the recombinant *E. coli* ER2566 strains carrying the plasmid pTXB1-CnAptA on LB medium in the presence of ampicillin (100 $\mu$g/ml), at 37 $^{\circ }$C until the optical density at 600 nm (OD600) for the culture reached 0.5–0.6, at which point 0.4 mM IPTG was added. We then incubated this culture at 22 $^{\circ }$C for another 10 h and harvested it by centrifugation at $2800{\times }g$, at 4 $^{\circ }$C.

### Purification of chitin-tagged CnAptA transaminase

We re-suspended the cell pellets from the previous centrifugation step in Tris-HCl buffer (20mM Tris-HCl, 0.5 M NaCl [pH 7.4]) and disrupted them using a Soniprep 150 MSE SANYO sonicator, at 4 $^{\circ }$C, at 30 s intervals for 30 min. We removed the insoluble fraction by centrifugation at $8400 \times g$ for 30 min. Then, we loaded the supernatant onto the chromatography column packed with the chitin resin (Poly-Prep^®^ prepacked columns, AG^®^ 1-X8, chloride form #731-1550), equilibrated with the same buffer at pH 8.4, according to the suppliers protocol. We induced the on-column cleavage of the CnAptA purified enzyme by adding Tris-HCl buffer containing 1,4-Dithiothreitol (DDT) (20 mM Tris-HCl, 0.5 M NaCl [pH 8.4]), 50 mM DTT). We then performed an elution of the un-tagged enzyme by adding 12 ml of Tris-HCl buffer (20 mM Tris-HCl, 0.5 M NaCl [pH 8.4]). Finally, we concentrated the eluted protein fraction and exchanged the elution buffer to a buffer containing 20 mM Tris-HCl, 0.05 M NaCl pH 8.4, using an Aminco Ultra-15 centrifugal filter unit (molecular mass cutoff, 50 kDa; Millipore). Hence, the target protein (CnAptA) was eluted from the column in a pure form (Fig. 15).

## Case in which $K= \textit{O}(1)$

In this Appendix, we provide the governing system in the depletion regime for the case with a strong reverse reaction when $K= \textit{O}(1)$, as the $t = \textit{O}(1)$ case proceeds in the same manner as when $K$ is small. Here, substrate depletion starts to affect the system at leading order when *t* becomes of $\textit{O}(1/\epsilon )$. To investigate this, we introduce $T = \epsilon t$, and we must also make the scalings $S_4= s_4/\epsilon$, and $S_6= s_6/\epsilon$. These scalings result in the system

71a $$\begin{aligned} \epsilon \dfrac{\mathrm {d} s_1}{\mathrm {d} T}&= - a\left( \epsilon v_{1}- V_{2}+ V_{-2}\right) , \end{aligned}$$

71b $$\begin{aligned} \dfrac{\mathrm {d} s_2}{\mathrm {d} T}&= - bv_{1}, \end{aligned}$$

71c $$\begin{aligned} \epsilon \dfrac{\mathrm {d} S_4}{\mathrm {d} T}&= \epsilon v_{1}-V_{2}+ V_{-2}, \end{aligned}$$

71d $$\begin{aligned} \epsilon \dfrac{\mathrm {d} s_5}{\mathrm {d} T}&= - c\left( V_{2}- V_{-2}\right) , \end{aligned}$$

71e $$\begin{aligned} \epsilon \dfrac{\mathrm {d} S_6}{\mathrm {d} T}&= V_{2}- V_{-2}, \end{aligned}$$

where the three reaction velocities are now defined as

72a $$\begin{aligned} v_{1}&= \left( \dfrac{s_1}{K_1^{(1)}+ s_1}\right) \left( \dfrac{s_2}{K_2^{(1)}+ s_2}\right) , \end{aligned}$$

72b $$\begin{aligned} V_{2}&= \left( \dfrac{S_4}{1 + S_4}\right) \left( \dfrac{s_5}{\delta + s_5}\right) , \end{aligned}$$

72c $$\begin{aligned} V_{-2}&= k^{(-2)}K\left( \dfrac{s_1}{K_{1}^{(-2)}+ s_1}\right) \left( \dfrac{S_6}{\epsilon K_{6}^{(-2)}+ S_6}\right) \left( \dfrac{S_6}{1 + KS_6}\right) . \end{aligned}$$

The matching conditions from the earlier timescale yield the following ‘initial conditions’

73 $$\begin{aligned} s_1(0) = 1, \quad s_2(0) = 1, \quad S_4(0) = 0, \quad s_5(0) = 1, \quad S_6(0) = 0. \end{aligned}$$

Naively taking the limit as $\epsilon \rightarrow 0$ in (34) to obtain a leading-order system would yield a duplication of information. To avoid this, we must form appropriate linear combinations of the governing equations in order to obtain enough information for a leading-order system. Using conserved quantities where possible, we obtain the following differential-algebraic system at leading-order

74a $$\begin{aligned} \dfrac{\mathrm {d} s_2}{\mathrm {d} T}&= - b\left( \dfrac{s_1}{K_1^{(1)}+ s_1}\right) \left( \dfrac{s_2}{K_2^{(1)}+ s_2}\right) , \end{aligned}$$

74b $$\begin{aligned} \dfrac{S_4}{1 + S_4}&= k^{(-2)}K\left( \dfrac{s_1}{K_{1}^{(-2)}+ s_1}\right) \left( \dfrac{S_6}{1 + KS_6}\right) , \end{aligned}$$

74c $$\begin{aligned} s_1+ aS_4&= 1, \end{aligned}$$

74d $$\begin{aligned} s_2+ bS_4+ bS_6&= 1, \end{aligned}$$

74e $$\begin{aligned} s_5+ cS_6&= 1. \end{aligned}$$

It is still possible to follow the analysis of Sect. 3.1 and rearrange this system into a single ODE, though this is too cumbersome to be particularly enlightening.

## Footnotes

1 Example KEGG classifications for this case would be EC 2.6.1.18 for the primary enzyme and EC 1.4.1.1 for the auxiliary enzyme.
